# Strategies for mitigating surface and interface defects in CIGS/perovskite tandem solar cells: a comprehensive review

**DOI:** 10.1186/s40580-026-00576-8

**Published:** 2026-09-11

**Authors:** Pravin S. Pawar, Rahul K. Yadav, Vishesh Manjunath, Anil V. Ghule, Gee Yeong Kim, Jaeyeong Heo

**Affiliations:** 1 Advanced Photovoltaics Research Center, Sustainable Energy Research Division, Korea Institute of Science and Technology, Seoul, 02792 Republic of Korea; 2 Department of Materials Science and Engineering and Optoelectronics Convergence Research Center, Chonnam National University, Gwangju, 61186 Republic of Korea; 3 Green Nanotechnology Laboratory, Department of Chemistry, Shivaji University, Kolhapur, 416004 India

**Keywords:** 2T tandem solar cells, CIGS, Perovskite, Surface defects, Interface engineering, Passivation, Photovoltaic efficiency

## Abstract

Two-terminal (2T) monolithic CIGS/perovskite tandem solar cells exploit complementary light-absorption characteristics to approach the theoretical Shockley–Queisser (SQ) efficiency limit (~ 45%), with recently reported highest power conversion efficiency (PCE) of 31.09% and a certified PCE of 30.57%. Nevertheless, surface and interface defects remain the critical loss mechanism, inducing non-radiative recombination, reducing open-circuit voltage (V_OC_), and accelerating degradation pathways. This review provides a systematic analysis of surface and interface defect chemistries and engineering strategies across 2T CIGS/perovskite architectures. We elucidate defect formation mechanisms at the CIGS/perovskite heterojunction and individual layer surfaces, encompassing point defects, grain boundaries, and structural imperfections, and their impact on device performance. Mitigation strategies are critically evaluated, including surface passivation, interface band-alignment engineering, compositional defect tolerance, and interconnection layer optimization. The coupled relationships between defect density, environmental degradation (moisture penetration, thermal cycling), and device reliability are analyzed systematically. Finally, we identify fundamental bottlenecks and research directions toward high efficiencies (≥ 32%), commercial scalability, and long-term operational stability in 2T CIGS/perovskite tandem solar cells.

**Graphical abstract:**

**Figure:**
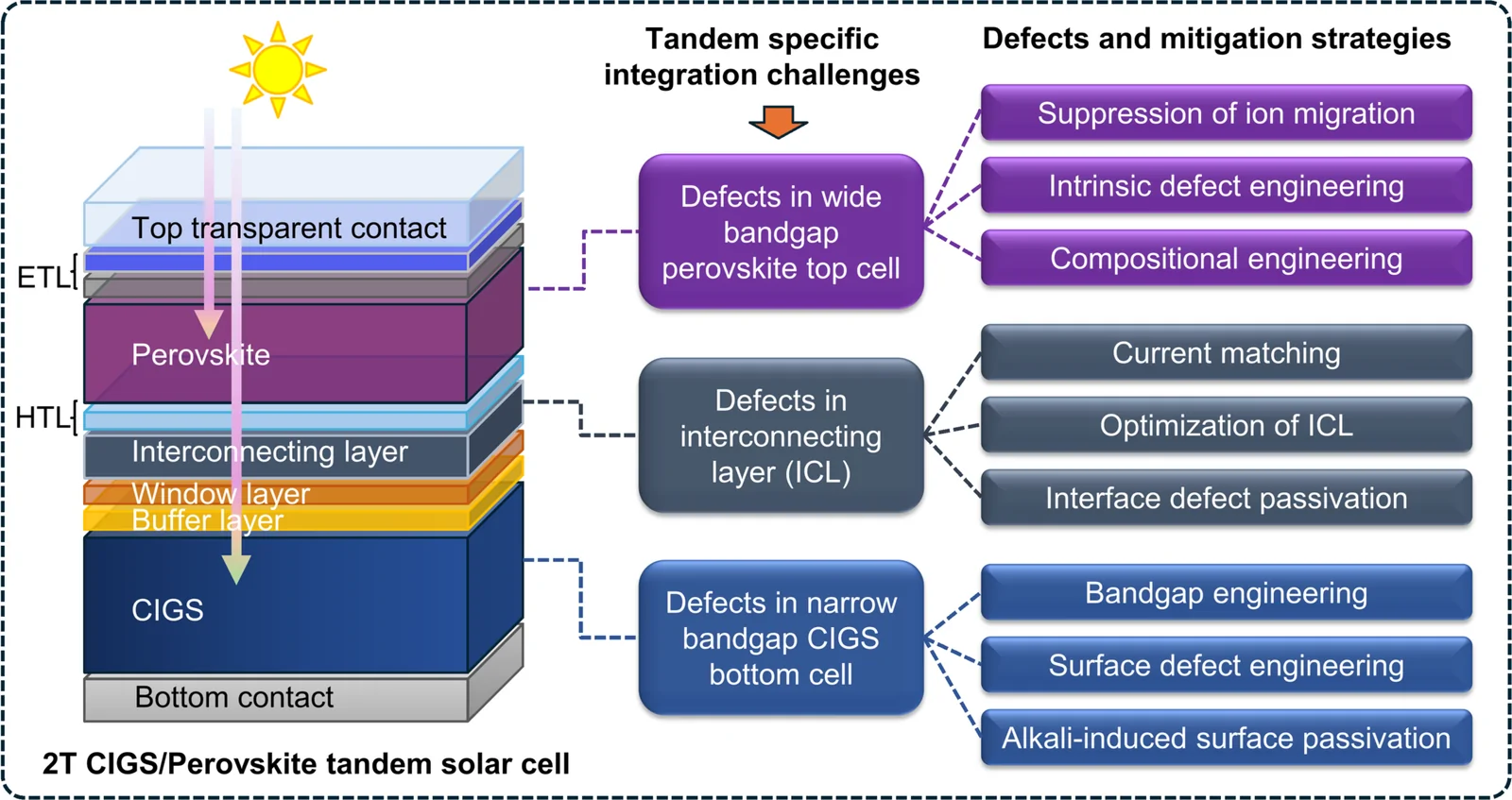

## Introduction

The rapid evolution of photovoltaic technologies toward high-efficiency, low-cost, and scalable solutions has positioned tandem solar cells as a leading strategy to surpass the fundamental power conversion efficiency (PCE) limits of single-junction devices [1–4]. In particular, monolithic integration of wide-bandgap (WBG) perovskite (*E*_g_ = ~ 1.63–1.68 eV) top cells with Cu(In,Ga)(S,Se)_2_ (CIGS) bottom cells (*E*_g_ = ~ 1.0–1.1 eV) has emerged as a highly promising pathway, combining the tunable optoelectronic properties of perovskites with technological maturity, stability, and infrared absorption capability of the CIGS [1, 5, 6]. Standard CIGS solar cells are n-on-p devices: a p-type CIGS absorber sits on the back contact, while an n-type buffer/window layer stack faces the illuminated side. This polarity is not a design choice but a fixed consequence of decades-optimized CIGS processing. It imposes an urgent, non-negotiable constraint on any two-terminal (2T) monolithic tandem built on top of it: because the two subcells share a single continuous recombination junction, the n-type front surface from CIGS must be paired with a hole-selective (p-type) contact from the perovskite side. Only this pairing enables efficient carrier recombination rather than accumulation and loss at the interface [7, 8]. This synergistic architecture enables more efficient utilization of the solar spectrum, with perovskites harvesting high-energy photons and CIGS absorbing lower-energy photons, thereby significantly enhancing PCE approaching the Shockley–Queisser (SQ) limit [9, 10]. Compared with Si/perovskite tandem solar cells, CIGS/perovskite tandems offer several intrinsic advantages that make them particularly attractive for next-generation monolithic tandem architectures. Unlike crystalline silicon, CIGS possesses a direct bandgap with a high absorption coefficient, enabling efficient near-infrared photon harvesting using substantially thinner absorber layers [5, 11]. Furthermore, the tunable bandgap of CIGS through Ga compositional engineering allows improved current matching with wide-bandgap perovskite top cells, thereby facilitating more effective spectral utilization and reducing optical losses [6]. CIGS also exhibits superior mechanical flexibility and compatibility with lightweight substrates, making CIGS/perovskite tandems highly suitable for flexible and building-integrated photovoltaic applications. In addition, the low-temperature fabrication routes and the compatibility of monolithic integration with CIGS can potentially reduce the thermal budget constraints commonly encountered in Si/perovskite tandem processing. These attributes collectively position CIGS/perovskite tandem solar cells as a promising alternative to conventional Si/perovskite tandems for achieving high-efficiency, lightweight, and scalable photovoltaic technologies [12]. Recent studies have demonstrated the potential of monolithic 2T CIGS/perovskite tandems, achieving a record PCE of 31.09% (30.57% certified) using a 1.01 eV CIGS bottom cell and 1.65 eV perovskite top cell, surpassing the previous 30.1% record and highlighting this architecture as a leading thin-film tandem technology [13, 14].

However, despite rapid progress, the practical realization of high-performance and stable CIGS/perovskite tandem devices remains fundamentally constrained by defects, particularly those localized at surfaces and interfaces [15]. Defects in photovoltaic materials have traditionally been interpreted in the framework of bulk properties, where their impact is mitigated by defect tolerance or compensated by a favorable electronic structure [16, 17]. CIGS, for instance, has long been considered a defect-tolerant semiconductor due to its ability to accommodate high concentrations of intrinsic point defects without catastrophic performance loss [18]. Similarly, metal halide perovskites exhibit a degree of defect tolerance arising from their soft lattice and benign defect energetics. This paradigm breaks down at surfaces and interfaces, where defect states are directly involved in charge separation, transport, and recombination processes [19]. In these regions, even relatively low defect densities introduce deep electronic states that function as efficient non-radiative recombination centers, thereby limiting open-circuit voltage (V_OC_), reducing carrier lifetimes, and degrading overall device PCE [20]. In the context of tandem solar cells, the role of surface and interface defects becomes even more critical. Unlike single-junction devices, tandem architecture involves multiple functional interfaces, including absorber/buffer interfaces, charge transport layers, recombination junctions, and interconnecting layers (ICL’s) [21]. Each of these interfaces introduces additional opportunities for defect formation, band misalignment, and interface recombination [22, 23]. Moreover, the integration of two fundamentally different material systems, CIGS and perovskites, adds further complexity due to differences in chemical composition, thermal stability, lattice structure, and processing conditions. These mismatches induce interface reactions, defect formation, and degradation pathways that remain absent in standalone devices. As a result, the performance and stability of tandem solar cells are governed primarily by the properties of the interfaces that connect the individual absorbers, rather than their intrinsic material quality [3]. A central aspect of defect behavior in tandem systems lies in the strong coupling between defect chemistry and electronic band alignment. At any heterointerface, the relative positions of the conduction and valence bands determine carrier transport barriers and recombination probabilities. Defects profoundly influence interface band alignment by introducing charged states, perturbing local electrostatic potentials, and generating compositional inhomogeneities at heterointerfaces [24]. Conversely, the electronic band structure itself governs defect formation energies, charge-state stability, and defect redistribution dynamics. Consequently, effective defect mitigation strategies must simultaneously integrate chemical passivation with electronic-structure engineering. In this context, interface-engineering approaches that concurrently optimize band alignment and suppress interface defect states have enabled substantial improvements in V_OC_ and fill factor (FF) in state-of-the-art devices [25, 26]. Equally important, surface and interface defects are inherently dynamic under operational conditions, evolving in response to illumination, electric-field bias, thermal stress, and ionic redistribution. In both CIGS and perovskite absorbers, defect populations evolve under illumination, electrical bias, and thermal stress [27, 28]. Processes such as ion migration, defect complex reconfiguration, and interface chemical reactions drive time-dependent variations in device performance, including hysteresis, metastability, and long-term degradation [29]. These effects become more pronounced in tandem architectures, where interactions between dissimilar material layers amplify instability mechanisms. Consequently, a comprehensive understanding of both the static properties of defects and their kinetics and evolution remains essential for the design of reliable and durable tandem solar cells. Recent advances in experimental characterization and computational modeling provide unprecedented insight into the nature of surface and interface defects in both CIGS and perovskite systems [30]. Techniques such as time-resolved photoluminescence (TRPL), Kelvin probe force microscopy (KPFM), atom probe tomography (APT), and synchrotron-based spectroscopy have enabled nanoscale mapping of defect distributions and electronic properties. At the same time, first-principles calculations and machine learning approaches have facilitated the prediction of defect energetics, diffusion pathways, and passivation mechanisms [31]. These developments have shifted the field from empirical optimization toward a more physics-driven understanding of defect behavior, opening new opportunities for rational design of interfaces [16, 32].

This review aims to provide a comprehensive and unified perspective on strategies for mitigating surface and interface defects in monolithic 2T CIGS/perovskite tandem solar cells. Although several reviews have summarized the development of CIGS/perovskite tandem solar cells, most primarily focus on device architecture and efficiency progress [33, 34]. In contrast, this review specifically examines surface and interface defects as the central factors governing the efficiency and stability of monolithic 2T CIGS/perovskite tandem solar cells. The review first discusses defect formation and mitigation strategies in the CIGS bottom cell and wide-bandgap perovskite top cell independently to establish the fundamental defect physics of each material system, followed by a dedicated discussion of tandem-specific interfaces, including recombination junctions and ICLs. By critically comparing recent defect-passivation and interface-engineering strategies reported for each component and the integrated tandem architecture, this review identifies the key scientific challenges and future research directions for realizing highly efficient, stable, and scalable CIGS/perovskite tandem photovoltaics. The discussion is structured to first establish the fundamental principles governing tandem architectures and their interfaces, followed by a detailed examination of defect formation and mitigation in each subcell. In this review, the first section focuses on the CIGS bottom cell, in which surface stoichiometry, alkali engineering, and secondary-phase formation play key roles in defect control. The second section addresses the perovskite top cell, emphasizing defect formation in wide-bandgap compositions and interface engineering with charge transport layers. The subsequent sections explore tandem-specific challenges, including interface defects at the CIGS/perovskite integration interface, recombination junction design, and the implications of defects for device stability and reliability. Finally, the review outlines current challenges, emerging opportunities, and future research directions aimed at achieving precise, scalable, and stable defect management in tandem photovoltaic systems. By integrating insights across materials, interfaces, and device architectures, this review establishes that surface and interface defects represent central determinants of performance and stability in monolithic 2T CIGS/perovskite tandem solar cells. Addressing these challenges requires a holistic framework that unifies materials design, process optimization, and interface engineering, thereby enabling the realization of next-generation tandem technologies with enhanced efficiency and long-term durability.

## Bottom cell (CIGS): surface and interface defects

### Surface defects in CIGS bottom cells

**Fig. 1:**
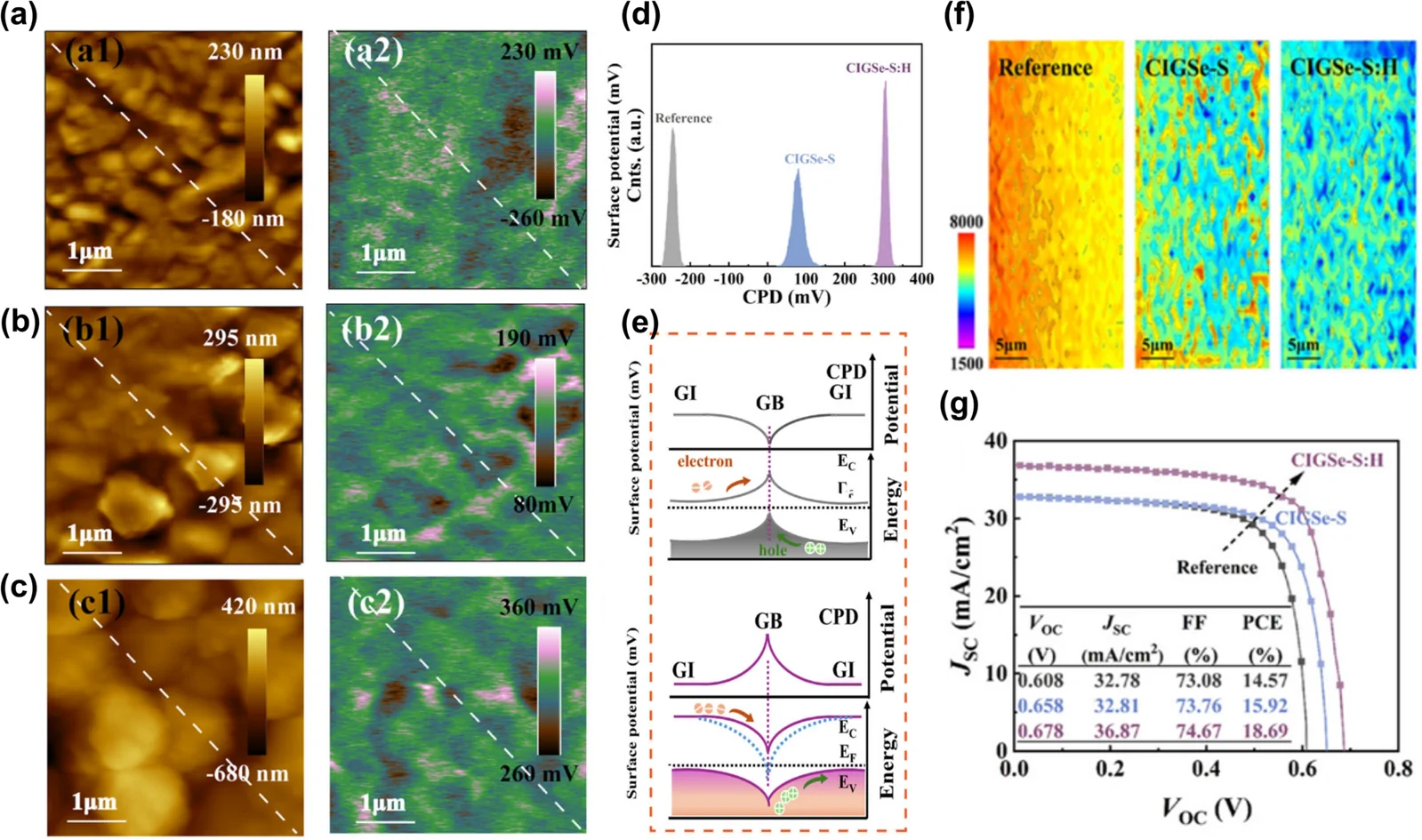
AFM surface topography and corresponding KPFM potential maps of **a** Reference, **b** CIGSe-S, and **c** CIGSe-S:H samples. **d** Surface contact potential difference (CPD) distributions. **e** Schematic illustration of the band structure and CPD variation near GBs. **f** Raman mapping images of the Reference, CIGSe-S, and CIGSe-S:H films. **g** Current density–voltage (J–V) characteristics of the corresponding devices. Reproduced with permission from Ref. [40]

Surface and interface defects in CIGS absorbers remain one of the primary barriers to achieving high-efficiency photovoltaic performance, particularly in wide-bandgap and tandem solar cell architectures [35, 36]. Although CIGS has long been regarded as a defect-tolerant semiconductor, recent experimental and theoretical studies have shown that this tolerance is largely limited to the bulk region of the absorber [37]. In contrast, surfaces and heterointerfaces are far more sensitive because band alignment, compositional inhomogeneity, and defect energetics collectively govern carrier recombination processes [38]. As a result, nonradiative recombination in these regions becomes a major source of voltage loss and performance degradation. Therefore, the research focus in advanced CIGS photovoltaics has gradually shifted from relying on intrinsic defect tolerance toward precise engineering and passivation of surface and interface defects, which are now recognized as key factors controlling carrier dynamics, V_OC_, and long-term device stability [35, 38, 39]. Figure 1 demonstrates the impact of surface defect passivation on the structural, electronic, and photovoltaic properties of CIGSe films. AFM and KPFM analyses (Fig. 1a–c) show that S and S:H treatments increase the surface potential while reducing local potential fluctuations, indicating effective suppression of surface defect states and enhanced grain-boundary (GB) passivation. The surface contact potential difference (CPD) distributions and schematic band diagrams (Fig. 1d–e) further reveal improved band bending and reduced carrier recombination near GBs. In addition, Raman mapping (Fig. 1f) confirms improved compositional uniformity after treatment. These modifications collectively lead to enhanced carrier transport and charge collection, as evidenced by the improved current density–voltage (J–V) characteristics and photovoltaic performance of the treated devices (Fig. 1g) [40].

### Defect tolerance versus defect control in CIGS

**Fig. 2:**
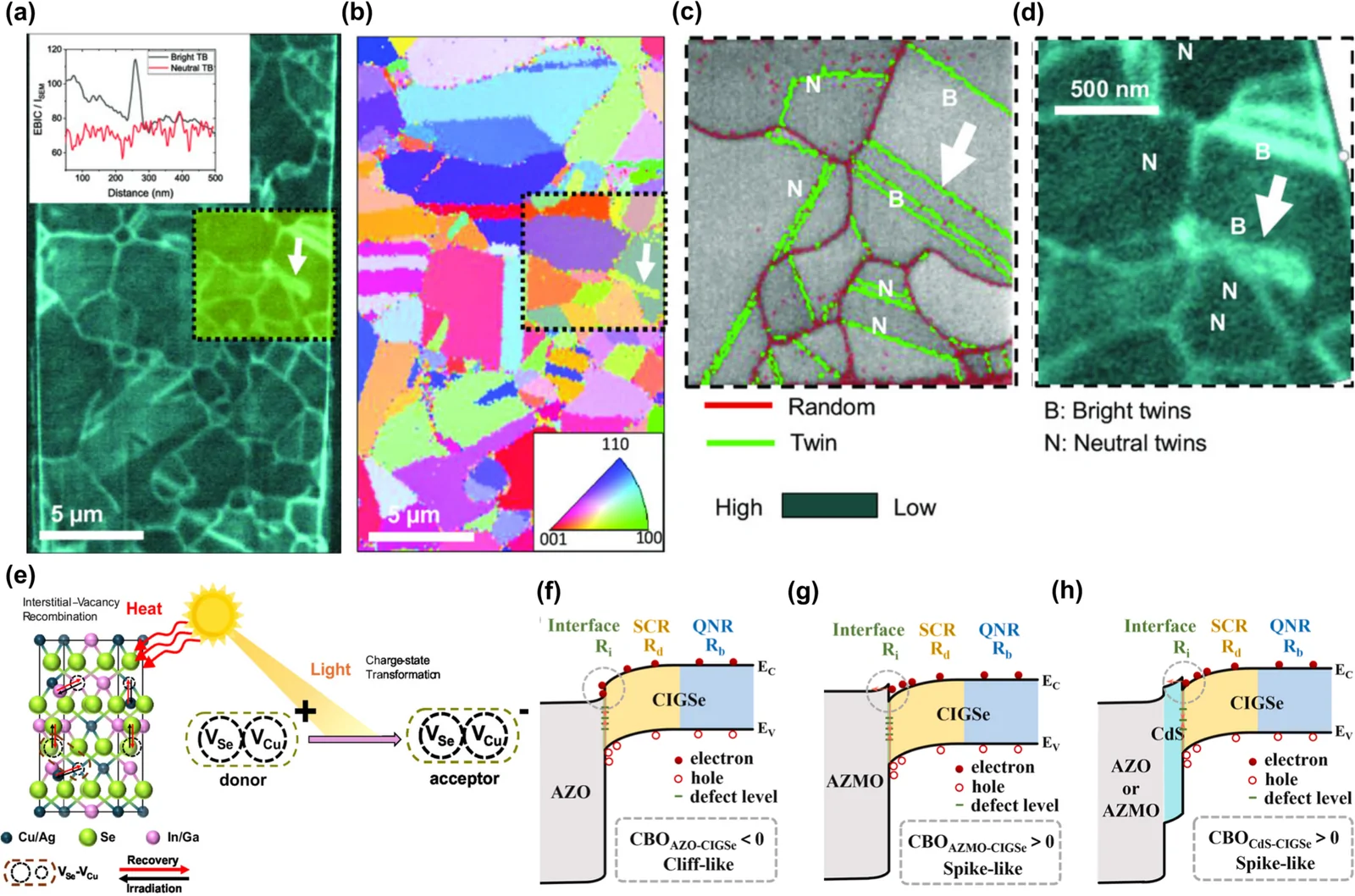
Correlative EBIC–EBSD investigations. **a** EBIC image acquired at 5 kV and 50 pA; inset shows current profiles of a bright and a neutral twin boundary (TB). **b** Corresponding EBSD inverse pole figure map from the identical region, with the outlined area indicating the TEM lamella location. **c** GB map of the TEM lamella obtained using TKD. **d** Magnified EBIC image of the TEM lamella region; the white arrow indicates a bright TB analyzed by HR-TEM. Reproduced with permission from Ref. [47]. **e** Schematic illustration of the HLS recovery mechanism in irradiated CIGS-based devices, highlighting thermally assisted interstitial–vacancy recombination and light-induced charge-state transformation of the V_Se_–V_Cu_ complex, with Ag incorporation enhancing defect passivation and lattice mobility. Reproduced with permission from Ref. [48]. Schematic band alignment diagrams at the p–n junction for **f** ZnO:Al/CIGSe, **g** Zn_0.96_Mg_0.04_O: Al/CIGSe, and (h) CdS/CIGSe. Reproduced with permission from Ref. [55]

The concept of defect tolerance in CIGS originates from its ability to accommodate high concentrations of intrinsic point defects without catastrophic degradation in photovoltaic performance [35, 37, 41]. Among these defects, copper vacancies (V_Cu_) play a particularly beneficial role by acting as shallow acceptors that stabilize p-type conductivity and facilitate efficient carrier transport [42, 43]. However, the defect landscape in CIGS is considerably more complex than initially assumed. In addition to benign shallow defects, deeper-level defects such as antisite defects (In_Cu_ and Ga_Cu_) and vacancy-related complexes can introduce mid-gap states that serve as efficient non-radiative recombination centers [44, 45]. The influence of these deep defects becomes increasingly critical in wide-bandgap CIGS absorbers, where higher gallium incorporation modifies defect formation energetics and shifts defect states deeper into the bandgap [46]. Consequently, recombination losses intensify, leading to larger V_OC_ deficits. These findings demonstrate that defect tolerance in CIGS is not an intrinsic or universal property, but rather a highly composition-dependent phenomenon governed by defect energetics, spatial distribution, and local chemical environments. Recent nanoscale studies have further revealed that defect distributions in CIGS are highly heterogeneous rather than uniformly distributed throughout the absorber [10]. Variations in carrier concentration and defect density across grains, GBs, and heterointerfaces create localized recombination “hot spots” that strongly influence macroscopic device behavior [10, 46]. In Cu-deficient CIS absorbers, for example, Cd incorporation into Cu vacancies during CdS deposition forms Cd_Cu_ donor defects, which induce surface inversion and create a beneficial buried p–n homojunction. This effect enhances carrier collection and contributes to improved short-circuit current density (J_SC_). Such observations highlight the critical role of local defect chemistry in determining charge separation and transport mechanisms. Figure 2 summarizes recent advances in nanoscale characterization and interface engineering of CIGSe solar cells. Correlative electron beam-induced current–electron backscatter diffraction/transmission Kikuchi diffraction (EBIC–EBSD/TKD) analyses (Fig. 2ab) provide direct evidence of the electrical activity associated with grain and twin boundaries, demonstrating that bright twin boundaries promote enhanced carrier collection compared with electrically neutral twins. Complementary high-resolution transmission electron microscopy (HR-TEM) investigations (Fig. 2cd**)** further reveal the local defect configurations and structural characteristics associated with these electrically active boundaries [47]. In addition, the schematic illustration in Fig. 2e describes the heat–light soaking (HLS) recovery mechanism in irradiated CIGSe devices, where thermally activated defect recombination and light-induced charge-state transformation of defect complexes improve p-type conductivity and defect passivation [48].

Together, these studies challenge the conventional reliance on average material properties and instead establish that local defect environments, particularly at surfaces and interfaces, govern recombination behavior and overall device performance. The limitations of defect tolerance become even more apparent in solution-processed CIGS systems, where incomplete crystallization and elevated defect densities lead to substantial recombination losses [6, 49]. These findings underscore the strong correlation between crystallization pathways, microstructural evolution, and the resulting electronic properties of the absorber. Complementary machine learning studies further support this perspective by identifying absorber defect density and interface quality as dominant factors controlling device efficiency [36, 50–53]. Collectively, these results indicate that future progress in CIGS photovoltaics requires a transition from passive reliance on intrinsic defect tolerance toward deliberate and active defect engineering strategies. Importantly, surface and interface properties in CIGS cannot be fully understood solely through equilibrium defect chemistry. Instead, they arise from a complex interplay among intrinsic point defects and defect complexes, GB reconstruction and termination, alkali impurity segregation, secondary-phase formation, and dynamic chemical reactions occurring during buffer-layer deposition [54]. Therefore, understanding and controlling surface defect formation requires a holistic and process-dependent approach that considers the coupled evolution of chemistry, microstructure, and electronic structure throughout device fabrication (Table 1).

**Table 1:** Intrinsic defects in CIGS absorbers and their electronic activity, formation energetics, and implications for carrier recombination and device efficiency

Defect type	Typical density (cm^−3^)	energy level	Device-level consequence	Ref
V_Cu_	10^16^–10^18^	Shallow acceptor	Beneficial: improves conductivity, but excess may enhance compensation	[36]
In_Cu_/Ga_Cu_ antisites	10^14^–10^16^	Deep donor/acceptor	V_OC_ deficit, increased ideality factor (*n* > 1.5)	[36]
V_Se_	Surface-enhanced (~ 10^15^–10^17^)	Donor-like, deep	V_OC_ ↓, FF ↓	[36]
(V_Se_–V_Cu_) complexes	Process-dependent	Mid-gap	Light soaking effects, hysteresis	[56]
Defect clusters	Highly variable	Mid-gap tail states	Band tailing, reduced quasi-Fermi level splitting	[57]

### Surface defect physics and competing mechanisms

The surface of CIGS absorbers constitutes a highly complex and dynamic region where structural, electronic, and chemical phenomena intersect [38, 47, 53, 58]. Unlike the bulk, where defect effects may be partially screened, surface and interface regions are directly involved in carrier separation and extraction, making them particularly sensitive to defect-induced recombination [53, 59]. Some of the Surface defects in CIGS are mentioned in Table 2. At the CdS/CIGS heterojunction, the interplay between defect states and band arrangement plays a decisive role in governing recombination dynamics. The conduction band offset (CBO) determines whether the interface forms a favorable “spike” or an unfavorable “cliff.” In the case of a spike-like alignment, a small energy barrier reduces electron backflow and suppresses recombination, whereas a cliff-type alignment facilitates carrier recombination by lowering the energetic barrier [60, 61]. The band alignment diagrams highlight the influence of ZnO: Al, AZMO, and CdS interface layers on conduction band offset, carrier transport, and interface recombination at the CIGSe heterojunction, as shown in Fig. 2f–h [55]. In general, increasing Ga incorporation raises the CIGS bandgap and shifts the conduction-band position, which can lead to unfavorable band alignment and enhanced interface recombination if not carefully engineered [46, 62]. While such effects are particularly relevant in high-Ga, wide-bandgap CIGS developed for single-junction devices or other tandem configurations, 2T CIGS/perovskite tandem solar cells typically employ lower-bandgap CIGS absorbers (~ 1.0–1.1 eV) to maximize near-infrared absorption and achieve efficient current matching with the wide-bandgap perovskite top cell. Nevertheless, careful optimization of interface band alignment and defect chemistry remains essential across all CIGS compositions to minimize non-radiative recombination and maximize device performance. Importantly, recent studies demonstrate that defect passivation alone is insufficient to mitigate these losses [62, 63]. Instead, defect chemistry and band alignment must be co-optimized, as interface defects and electronic structure are intrinsically coupled. Advanced buffer-layer strategies, such as Zn(O, S)/CdS bilayers, exemplify this approach by simultaneously tailoring band alignment and reducing interface defect density, thereby significantly improving V_OC_ [56, 64]. In addition to static defect states, the CIGS surface exhibits pronounced metastability. Defect populations evolve under illumination, electrical bias, and thermal stress, driven by the reconfiguration of defect complexes such as V_Se_–V_Cu_ [52, 53, 65, 66]. This dynamic behavior governs carrier lifetimes, recombination pathways, and overall device stability. Notably, radiation studies demonstrate that certain defect configurations undergo partial reversal through thermal and light-assisted annealing, indicating a self-healing response that distinguishes CIGS from many other photovoltaic materials. GBs further complicate the surface defect landscape. While they have traditionally been considered detrimental, recent studies reveal that not all GBs contribute equally to recombination [67, 68]. In particular, Σ3 twin boundaries exhibit electronic properties comparable to the bulk and do not introduce significant recombination activity. In contrast, random GBs often host defect states that act as efficient recombination centers. This distinction underscores the importance of selective defect mitigation strategies that target specific boundary types, rather than treating all GBs uniformly [69, 70]. Finally, the rear interface between CIGS and the back contact has emerged as an equally important contributor to recombination losses. Defects at the Mo/CIGS interface, together with suboptimal band alignment, limit carrier extraction and introduce additional recombination pathways [71]. Recent advances in rear interface engineering, including the incorporation of two-dimensional materials such as WSe_2_, demonstrate that back-contact design plays a decisive role in governing carrier dynamics and overall device efficiency [35, 36, 62, 69, 72–74].

**Table 2:** Surface and GB defects in CIGS and their electronic characteristics and influence on device performance

Location	Defect characteristics	Electronic behavior	Performance impact		Ref
Surface (Cu-poor, OVC-rich)	Ordered vacancy compounds	Bandgap widening (~ 0.1–0.3 eV)	Reduced recombination if optimized; increased resistance if excessive	[63]	
Surface (Se-deficient)	High V_Se_ concentration	Strong recombination	V_OC_ ↓ (~ 100–200 mV)	[36]	
Grain boundaries (Cu-poor)	Cu depletion + alkali segregation	Downward band bending	Enhances carrier separation	[62]	
Grain boundaries (Cu-rich)	Cu accumulation	Recombination-active	FF and V_OC_ ↓	[62]	
Twin boundaries (Σ3)	Ordered structure	Electrically benign	Minimal recombination	[47]	
Nanoscale inhomogeneity	Spatial defect variation	Local recombination hotspots	Efficiency variability, FF ↓	[75]	

### Alkali-induced surface passivation: success and uncertainty

The incorporation of alkali elements into CIGS absorbers represents one of the most significant advances in thin-film photovoltaics, contributing substantially to the continuous improvement in device efficiency over the past decades [76–78]. Despite its widespread adoption, alkali engineering remains mechanistically complex, particularly regarding the passivation of surface and interface defects. Consequently, understanding alkali-induced passivation requires not only evaluating its beneficial effects on device performance but also systematically examining the associated incorporation pathways, spatial distributions, and potential trade-offs [79]. At the microscopic level, alkali species modify the defect chemistry of CIGS by interacting with intrinsic defect sites and altering the local electrostatic environment [80–82]. Among the alkali elements, sodium (Na) has been the most extensively studied because of its natural diffusion from soda-lime glass substrates and its deliberate incorporation during growth. In the bulk absorber, Na promotes p-type conductivity by enhancing shallow acceptor formation and increasing carrier concentration [78, 81]. Simultaneously, it influences grain-growth kinetics, leading to larger grains and reduced GB density. Near GBs and surfaces, Na can suppress recombination-active states by passivating dangling bonds and reducing defect densities, thereby improving the FF and overall device efficiency [71, 83].

**Fig. 3:**
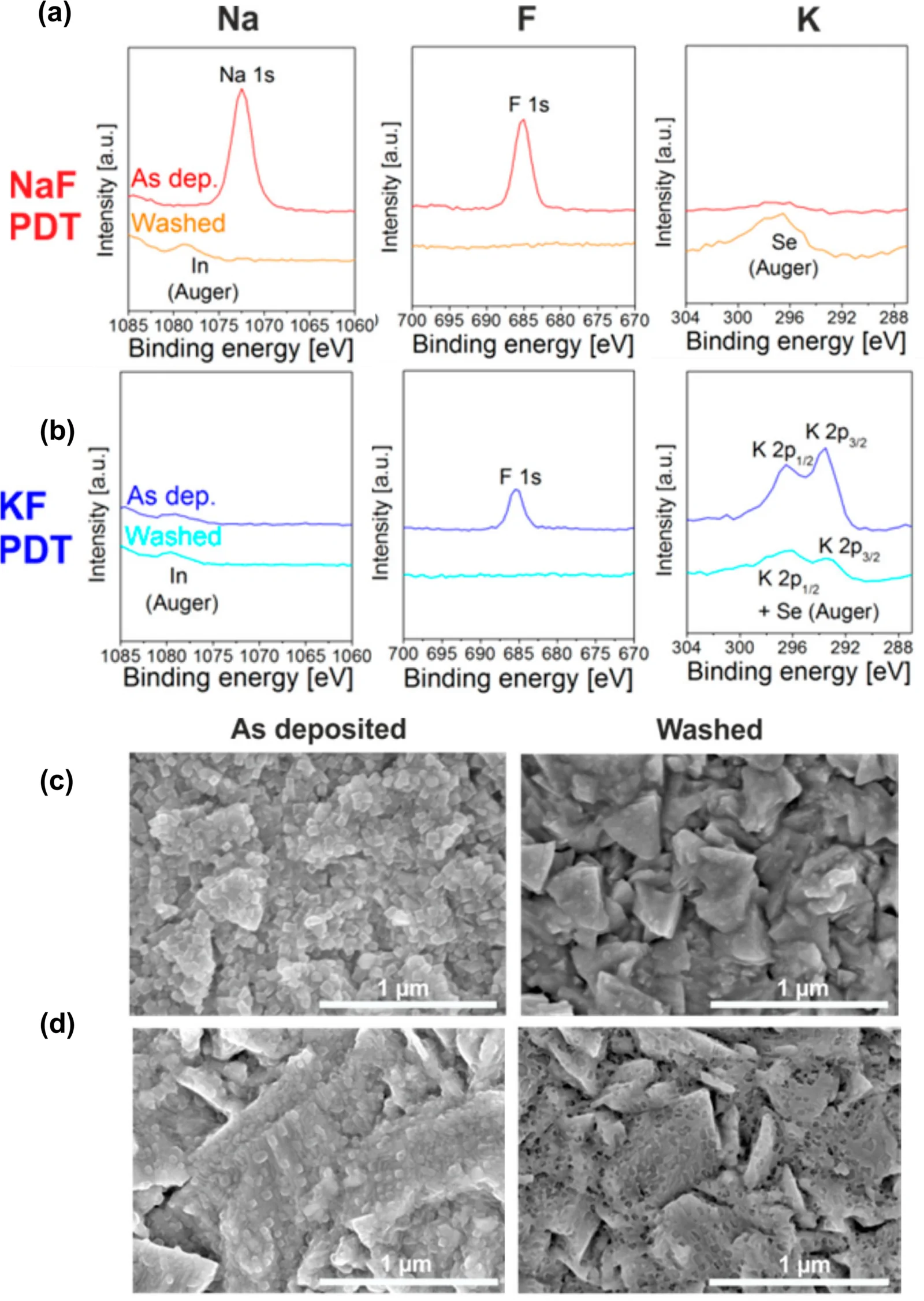
XPS survey spectra highlighting the binding energy regions of the Na 1s, F 1s, and K 2p peaks for CIGS absorbers subjected to **a** NaF PDT and **b** KF PDT. Spectra were acquired from as-deposited samples (NaF, KF) and from samples treated with a diluted ammonia solution (NaF (washed), KF (washed)). Corresponding SEM micrographs in **c** and **d** compare the CIGS surface morphology before and after the washing process. Reproduced with permission from Ref. [84]

Figure 3 illustrates the surface chemical and morphological evolution of CIGS absorbers subjected to NaF and KF post-deposition treatments (PDTs), both before and after washing in a diluted ammonia solution. The X-Ray Photoelectron Spectroscopy (XPS) spectra shown in Figs. 3a-b reveal distinct differences in the chemical stability and surface distribution of alkali-related species introduced by the two PDT processes [84]. In the NaF-treated absorber, pronounced Na 1s and F 1s peaks are clearly observed in the as-deposited condition, confirming the formation of Na-containing fluoride species on the CIGS surface. Following ammonia washing, both signals are significantly reduced or nearly eliminated, indicating that these species are primarily confined to the outermost surface and can be readily removed during cleaning. No detectable K-related signal is observed in the NaF-treated sample, and the weak feature near the K 2p region originates from the Se Auger transition rather than potassium incorporation. In contrast, the KF-treated absorber exhibits a distinct K 2p doublet together with a pronounced F 1s peak in the as-deposited state, confirming successful incorporation of potassium-containing surface species during KF PDT. After ammonia washing, the F signal almost completely disappears, whereas weak K-related features remain detectable. This observation suggests that a fraction of the incorporated K species is more strongly bonded or diffused into the near-surface region of the absorber. The persistence of K after washing is consistent with previous reports indicating that KF PDT induces stable surface reconstruction, defect passivation, and compositional modification near the CIGS/CdS interface.

The scanning electron microscopy (SEM) micrographs shown in Fig. 3c –d further demonstrate the strong influence of the washing process on surface morphology [84]. Prior to washing, both PDT-treated surfaces are covered with fine particulates and secondary-phase-like agglomerates, commonly attributed to alkali-fluoride reaction products formed during PDT. After ammonia treatment, these surface features are largely removed, exposing larger faceted CIGS grains and producing a cleaner surface morphology. The KF-treated surface, in particular, exhibits a porous or partially etched texture after washing, implying partial dissolution of alkali-rich secondary phases. These observations indicate that ammonia washing effectively removes loosely bound surface compounds while preserving the underlying chalcopyrite structure. Collectively, the XPS and SEM results demonstrate that alkali-fluoride PDTs generate chemically distinct surface environments and that post-treatment cleaning plays a critical role in controlling the interface chemistry prior to buffer-layer deposition.

**Fig. 4:**
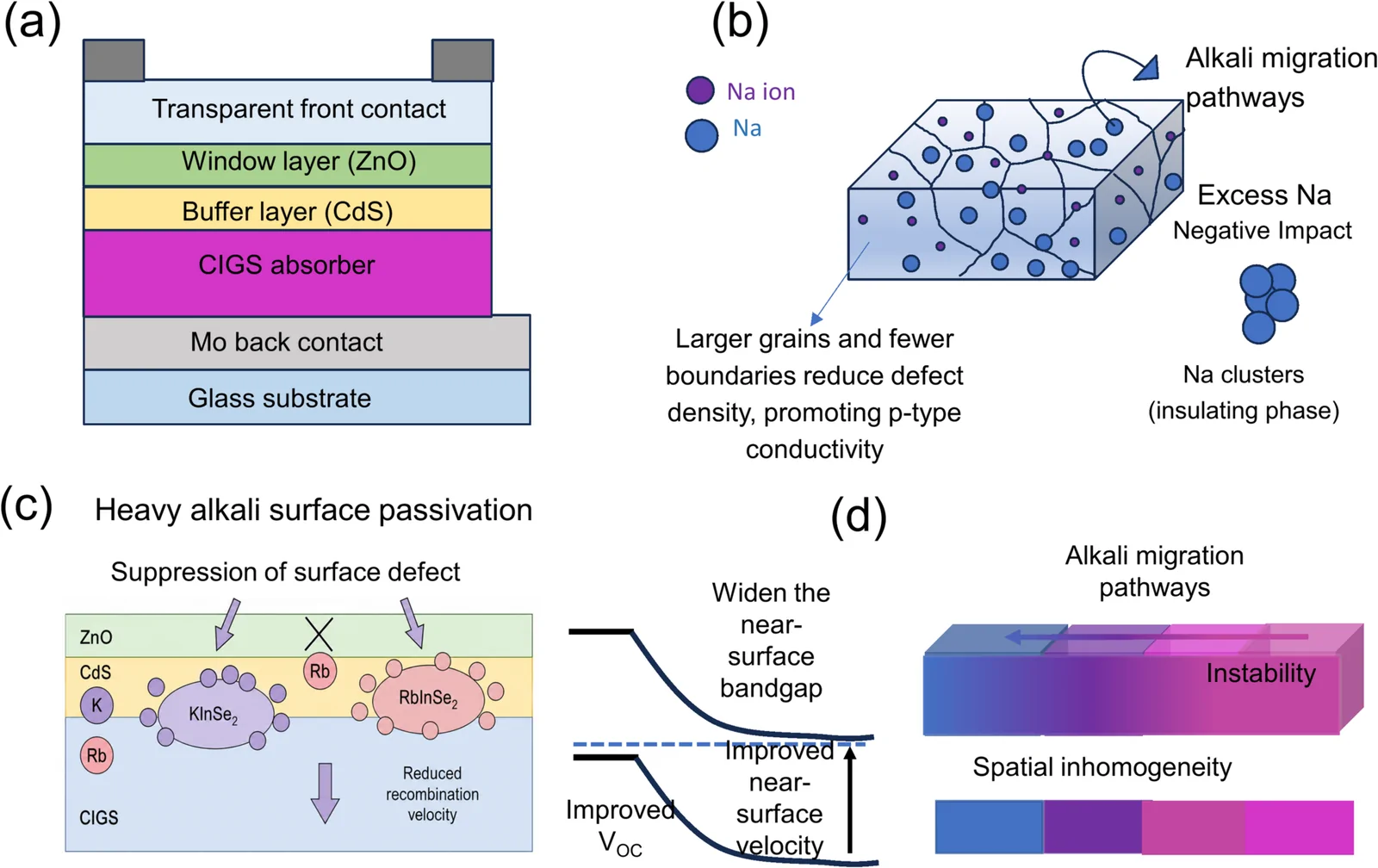
Schematic illustration of alkali engineering and surface passivation mechanisms in CIGS solar cells. **a** Schematic architecture of a CIGS solar cell. **b** Role of Na incorporation in modifying grain growth, passivating defects, and inducing compositional gradients within the absorber. **c** Heavy alkali surface treatment using K and Rb, showing the formation of alkali–selenide phases, suppression of surface defects, widened near-surface bandgap, and reduced interface recombination, leading to improved V_OC_. **d** Alkali migration pathways, spatial inhomogeneity, and multi-alkali strategies for optimizing defect passivation and interface stability

Although alkali incorporation provides substantial benefits, its effectiveness strongly depends on concentration, spatial distribution, and chemical stability. Figure 4a schematically illustrates a substrate configuration suitable for tandem extension, highlighting the importance of controlled alkali incorporation in advanced device architectures. Excessive Na incorporation can lead to the formation of insulating secondary phases and disturb compositional uniformity by suppressing In–Ga interdiffusion, thereby generating compositional gradients that degrade band alignment and introduce additional recombination pathways, as illustrated in Fig. 4b [85]. Consequently, alkali incorporation should not be regarded as universally beneficial; rather, Na acts as a process-dependent modifier of defect energetics, microstructure, and compositional evolution. In comparison with Na, heavier alkali elements such as potassium (K) and rubidium (Rb) exhibit a stronger tendency toward surface localization. These elements are typically introduced through PDT processes, where they accumulate near the absorber surface and form alkali–In–Se compounds such as KInSe_2_ and RbInSe_2_, as illustrated in Fig. 4c [2]. Such phases effectively suppress surface defect states, particularly antisite- and vacancy-related defects, thereby reducing interface recombination velocity and enhancing the V_OC_ [86, 87]. This behavior highlights an important principle of alkali engineering: different alkali species perform site-specific roles governed by their chemical reactivity, diffusion behavior, and incorporation pathway rather than acting uniformly throughout the absorber. Beyond defect passivation, alkali incorporation also influences the electronic band structure and surface energetics of CIGS absorbers [88]. Alkali-induced widening of the near-surface bandgap can create favorable energy gradients that promote carrier separation and suppress interface recombination. Such localized bandgap modification is particularly beneficial for mitigating interface recombination associated with Ga-rich surface regions without compromising bulk carrier collection [75]. However, extending the bandgap widening throughout the entire CIGS absorber would reduce near-infrared photon absorption and degrade current matching with the wide-bandgap perovskite top cell in monolithic tandem architectures. Therefore, localized bandgaps, in which a low-bandgap bulk absorber is combined with widened bandgaps near the front and rear interfaces, represent a more effective strategy for simultaneously achieving high J_SC_, enhanced V_OC_, and reduced interface recombination.

### Bandgap grading strategies for 2T CIGS/perovskite tandem solar cells

Another important strategy for optimizing 2T CIGS/perovskite tandem solar cells is intentional bandgap grading within the CIGS bottom absorber. Unlike high-efficiency single-junction CIGS devices, which often employ relatively wider bandgaps to maximize the V_OC_, tandem architectures require a lower bulk bandgap (~ 1.0–1.1 eV) to efficiently harvest near-infrared photons transmitted through the wide-bandgap perovskite top cell and achieve optimal current matching between the two subcells. Consequently, the objective of bandgap engineering in tandem CIGS absorbers is not to increase the bandgap throughout the entire absorber, but rather to maintain a narrow-bandgap bulk while introducing localized bandgap widening near the front and rear interfaces through controlled Ga and/or S compositional grading [89, 90]. A double-graded bandgap profile is therefore commonly employed, where the bandgap remains narrow in the absorber bulk while increasing toward both interfaces. The wider bandgap near the front interface suppresses interface recombination by creating an electron-reflecting field and improving conduction-band alignment with the buffer layer, thereby enhancing the V_OC_. Similarly, rear bandgap grading reduces carrier recombination at the back contact, promotes efficient minority-carrier collection, and further contributes to voltage enhancement. Since these wider-bandgap regions are confined to relatively thin interface zones, the absorber retains its strong near-infrared absorption capability, allowing high J_SC_ to be maintained while simultaneously minimizing recombination losses. Beyond improving carrier transport and interface energetics, compositional grading also influences the local defect formation energy, carrier distribution, and the spatial activity of recombination centers throughout the absorber [89, 91]. In monolithic CIGS/perovskite tandem devices, appropriately designed grading profiles can further facilitate favorable energy-level alignment at recombination junctions and interconnecting layers, thereby improving carrier selectivity and reducing voltage losses across the tandem structure [92]. The effectiveness of bandgap grading, however, depends not only on the intended compositional profile but also on the CIGS deposition process. In particular, the achievable grading intensity is strongly influenced by the processing temperature. Lower-temperature fabrication better preserves steep Ga concentration gradients by suppressing elemental interdiffusion, enabling sharper bandgap transitions at the interfaces. Conversely, higher-temperature growth promotes Ga diffusion within the absorber, resulting in smoother grading profiles and limiting the realization of strong bandgap gradients. Excessive compositional gradients or poorly controlled grading may additionally introduce strain, elemental segregation, and spatially non-uniform defect distributions that degrade electronic homogeneity and long-term device stability [93]. Therefore, in monolithic CIGS/perovskite tandem solar cells, bandgap grading should be regarded not merely as a means of enhancing optical performance, but as a comprehensive strategy for simultaneously optimizing current matching, band alignment, defect chemistry, carrier transport, and interface recombination.

To address these challenges, multi-alkali approaches incorporating combinations of Na, K, Rb, and even Cd are increasingly being explored to exploit complementary functionalities. In such strategies, Na primarily improves bulk conductivity and grain growth, whereas heavier alkalis provide targeted surface and interface passivation, as illustrated in Fig. 4d. For example, Rb enrichment near the front interface can promote the formation of a thin RbInSe_2_ layer, which improves band alignment and suppresses interface recombination, thereby supporting enhanced J_SC_ [2, 94]. Nevertheless, achieving precise alkali control remains challenging because it requires balancing defect passivation, secondary-phase formation, compositional uniformity, and long-term stability. Overall, alkali-induced passivation in CIGS absorbers is both highly effective and intrinsically complex. Its effectiveness arises from the simultaneous modification of defect chemistry, band alignment, and microstructure [6]. At the same time, limitations associated with spatial inhomogeneity, secondary-phase formation, and alkali migration highlight the need for more controlled and predictive incorporation strategies. Future progress will therefore depend on developing site-selective, concentration-controlled, and dynamically stable alkali engineering approaches that transform alkali incorporation from an empirical optimization technique into a reliable and precisely tunable defect-management strategy.

### Secondary phases and surface stoichiometry

**Fig. 5:**
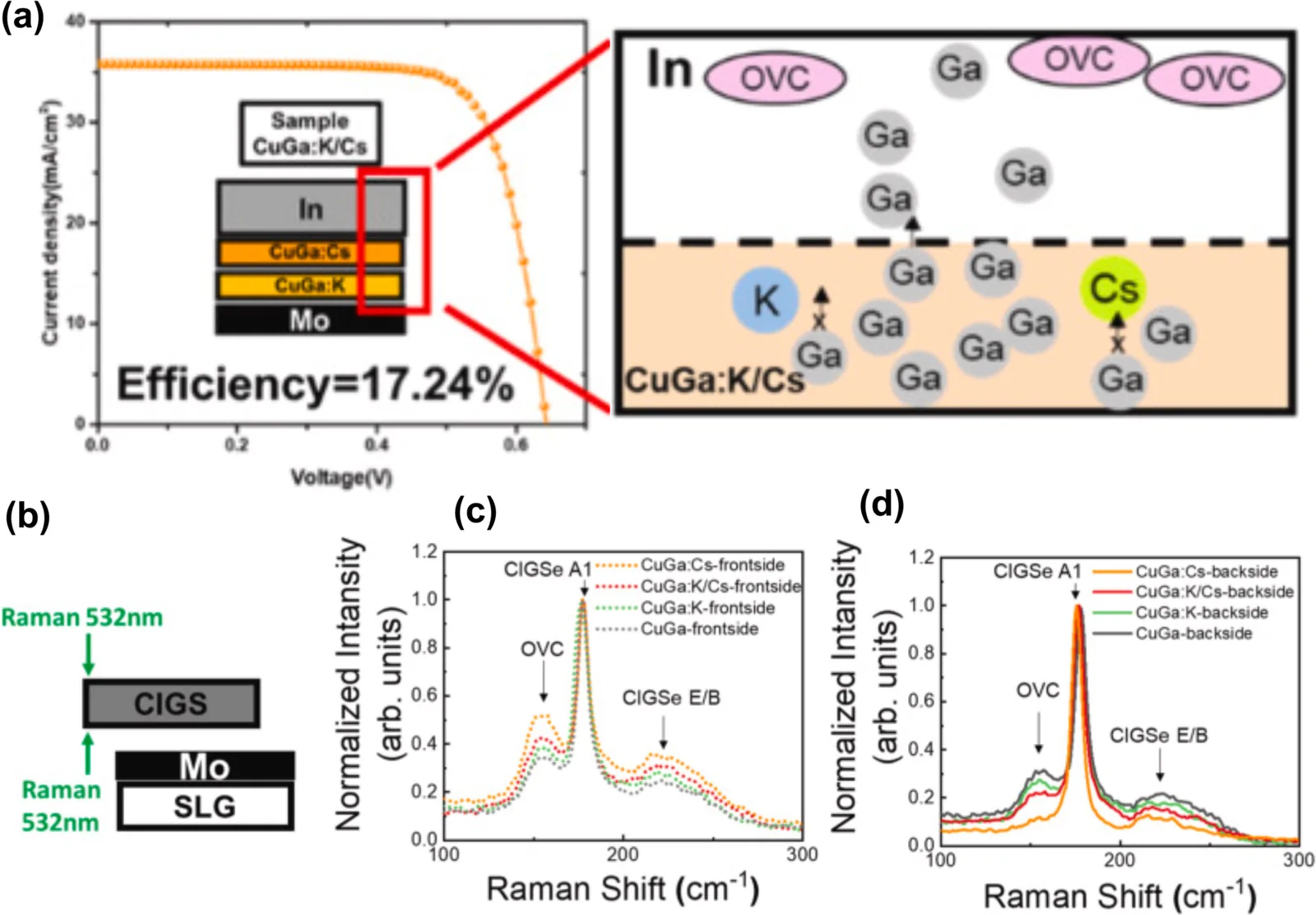
**a** J–V characteristics and schematic device structure of the CuGa: K/Cs-modified CIGS solar cell, demonstrating enhanced photovoltaic performance with a PCE of 17.24%. **b** Schematic illustration of the CuGa: K/Cs interface modification mechanism, showing suppression of OVCs and regulation of Ga distribution through K and Cs incorporation. **c** Schematic representation of front-side and back-side Raman measurement configurations for CIGS/Mo/SLG structures using a 532 nm excitation source. **d** Front-side and back-side Raman spectra of CIGSe films with different alkali treatments, highlighting variations in OVC-related features and CIGSe vibrational modes associated with defect passivation and compositional modification. Reproduced with permission from Ref. [99]

Surface stoichiometry in CIGS absorbers plays a critical role in determining the formation, distribution, and electronic activity of defects, particularly at the heterojunction interface where recombination losses are most severe [73]. Unlike bulk defect engineering, which primarily focuses on intrinsic point defects, surface stoichiometry introduces additional complexity by influencing secondary-phase formation, compositional gradients, and local band structure modifications [57, 95]. Consequently, precise control of surface composition has emerged as a key strategy for simultaneously optimizing defect passivation and interface energetics. Under Cu-poor growth conditions, commonly employed in high-efficiency CIGS devices, the absorber surface transitions into a Cu-deficient regime that promotes the formation of ordered vacancy compounds (OVCs), such as CuIn_3_Se_5_ [53, 96]. These phases contain a reduced Cu concentration and a high density of ordered vacancies, which significantly modify the local electronic structure. Importantly, thin OVC layers are generally beneficial because they suppress deep-level defect formation and create a widened surface bandgap [97, 98]. This bandgap widening induces a built-in electric field that promotes carrier separation and reduces recombination probability. In this context, OVC formation represents an intrinsic self-passivation mechanism arising from controlled non-stoichiometry (Fig. 5). However, its beneficial impact depends strongly on thickness, composition, and uniformity. A thin OVC layer enhances device performance, whereas excessive formation generates insulating regions that impede carrier transport. This behavior underscores the critical balance required in surface stoichiometry control, where both insufficient and excessive compensation degrade device performance [98].

The interaction between alkali incorporation and surface stoichiometry further increases the complexity of surface defect regulation. Alkali treatments facilitate the formation of secondary phases that act as effective passivation layers by modifying both the chemical composition and electronic structure of the surface [100, 101]. These modifications reduce defect density, improve band alignment, and suppress recombination losses. However, the formation of alkali-induced phases is highly sensitive to processing conditions. Uncontrolled growth can lead to phase segregation or electrically inactive regions that introduce additional transport barriers [101]. Accordingly, alkali-induced secondary phases enhance performance only when precisely regulated to prevent the introduction of new defects or transport barriers. In parallel, compositional engineering strategies provide additional pathways for controlling surface stoichiometry and defect behavior [102]. The incorporation of silver, for example, has been shown to reduce antisite defect formation by modifying the local chemical environment and stabilizing favorable defect configurations. This results in improved crystallinity and reduced recombination. Similarly, antimony-assisted growth strategies influence crystallization pathways, promoting larger grain formation and suppressing the formation of defective interface layers [102]. These approaches demonstrate that surface stoichiometry is not merely a passive consequence of growth conditions, but rather an actively tunable parameter that enables simultaneous optimization of structural and electronic properties. Another important factor is elemental interdiffusion at the heterointerface. Controlled diffusion of elements such as Cd or Zn from the buffer layer into the absorber can provide beneficial defect compensation, reduce band tailing, and enhance carrier collection efficiency [103]. Conversely, excessive interdiffusion disrupts surface compositional balance and can generate new defect states that increase recombination losses. This dual behavior highlights the importance of carefully controlling interface reactions during device fabrication to maintain favorable interface energetics. In WBG CIGS absorbers, surface stoichiometry becomes even more critical because defect energetics are highly sensitive to composition. Sulfur incorporation, for instance, modifies both the absorber bandgap and defect formation energies, enabling improved band alignment and reduced recombination losses [104].

Overall, surface stoichiometry in CIGS absorbers is a highly sensitive, multifaceted parameter that governs defect formation, passivation, and electronic structure. The formation of secondary phases, while often beneficial, must be carefully managed to avoid unintended consequences. Future progress in this area will require a more nuanced understanding of the relationships between composition, phase formation, and electronic properties, enabling the development of precise, spatially controlled stoichiometry for optimal defect management and interface performance.

However, sulfur-induced compositional fluctuations and sulfur-related defects can also introduce additional recombination pathways if not carefully controlled. Thus, the advantages of bandgap engineering in wide-bandgap CIGS are intrinsically linked to the ability to precisely regulate surface composition and defect formation. Figure 5 highlights the impact of alkali-assisted interface engineering on defect suppression and electronic quality in CIGS absorbers. The incorporation of K and Cs at the Cu and Ga interface significantly improves device performance, resulting in a PCE of 17.24%, as evidenced by the J–V characteristics [99]. The schematic illustration suggests that alkali incorporation regulates Ga redistribution while suppressing the formation of detrimental OVC-related defects, thereby reducing recombination losses. Complementary front-side and back-side Raman analyses further confirm modifications in OVC-related vibrational features and CIGSe lattice modes, indicating improved compositional uniformity and enhanced defect passivation throughout the absorber layer. Overall, surface stoichiometry in CIGS absorbers represents a highly sensitive and multifaceted parameter that governs defect formation, passivation behavior, interface energetics, and carrier transport. Although secondary-phase formation and compositional modification can substantially improve device performance, these effects remain strongly dependent on precise control of composition and interface reactions. Future progress will therefore require a deeper understanding of the relationships among stoichiometry, phase evolution, defect energetics, and electronic structure, ultimately enabling spatially controlled surface engineering strategies for next-generation high-efficiency CIGS solar cells.

### Implications for 2T CIGS/perovskite tandem architectures

The control of surface and interface defects in CIGS becomes even more critical in tandem solar cell architectures, where the performance of the bottom cell directly determines the efficiency of the overall device [1]. In 2T CIGS/perovskite tandem solar cells, the CIGS bottom absorber must possess a narrow bandgap of approximately 1.0–1.1 eV to efficiently harvest the near-infrared photons transmitted through the wide-bandgap perovskite top cell while providing sufficient photocurrent for current matching [105]. Although the theoretical optimum bottom-cell bandgap for an ideal two-junction tandem is approximately 0.96 eV, CIGS is one of the few mature thin-film photovoltaic technologies capable of approaching this value through Ga compositional engineering, with CIGS exhibiting a bandgap close to 1.0 eV. Furthermore, compared with crystalline silicon (*E*_g_ = 1.12 eV), the tunable bandgap of CIGS provides significantly greater flexibility for tandem device optimization. Despite these advantages, pure CIGS absorbers generally suffer from severe interfacial recombination and a large V_OC_ deficit, limiting their practical performance [46]. Moreover, defects located at the CIGS heterointerface and tandem interconnecting layers introduce non-radiative recombination pathways that propagate through the tandem stack, reducing carrier extraction efficiency, lowering photovoltage, and ultimately constraining the overall PCE [46, 104]. Recent studies have demonstrated that carefully engineered low-bandgap CIGS absorbers (≈ 1.01 eV) incorporating optimized front and back Ga grading can largely preserve the strong near-infrared absorption of CIGS while substantially enhancing V_OC_, yielding certified efficiencies exceeding 20%, making these absorbers highly attractive bottom cells for tandem applications. Equally important, excessive Ga incorporation (bandgap > 1.2 eV) has been shown to increase surface recombination and weaken CIGS/CdS interface passivation, whereas lower-Ga absorbers exhibit superior interface passivation and reduced recombination losses. These findings highlight that precise control of bandgap grading, interface chemistry, and defect passivation is essential for simultaneously minimizing voltage losses, maintaining favorable band alignment at both the CIGS/buffer interface and the tandem recombination junction, and promoting efficient charge transport in tandem devices [73].

Beyond bandgap optimization, the intrinsic properties of CIGS, including its excellent environmental stability, radiation resistance, and partial defect self-healing capability, make it an attractive bottom-cell technology for next-generation tandem photovoltaics. More importantly, the evolution of CIGS research from empirical process optimization toward a physics-based understanding of defect chemistry, interface energetics, and band alignment has established design principles that extend well beyond CIGS itself. Strategies such as defect passivation, interface engineering, compositional control, and bandgap grading are directly applicable to overcoming analogous limitations in perovskite-based tandem solar cells. Taken together, these studies demonstrate that surface and interface defects are not merely secondary imperfections but fundamental determinants of carrier recombination, energy-band alignment, and long-term device stability. Consequently, continued progress in 2T CIGS/perovskite tandem solar cells will depend on predictive, multiscale control of defects, interfaces, and band profiles, enabling the rational design of tandem photovoltaic devices with higher efficiency and enhanced operational durability [104].

## Defect formation and mitigation in wide-bandgap perovskite top cells

The WBG perovskites (E_g_ ~ 1.65–1.68 eV) are essential for achieving optimal current matching in monolithic CIGS/perovskite tandem solar cells [106]. However, their performance is mainly constrained by defect-induced non-radiative recombination and poor operational stability. WBG compositions are particularly susceptible to enhanced ion migration and halide segregation, inducing bandgap inhomogeneity and pronounced V_OC_ losses [107–109]. The optimum bandgap of the perovskite top absorber depends on the bandgap of the bottom cell. Thus, the susceptibility to photo-induced phase segregation varies with the tandem architecture. Monolithic Si/perovskite tandems typically employ top cell bandgaps of ~ 1.65–1.80 eV, whereas all-perovskite tandems generally require even wider-bandgap absorbers (~ 1.75–1.90 eV or higher), necessitating increased Br incorporation. In contrast, CIGS/perovskite tandems can utilize comparatively lower top-cell bandgaps (~ 1.60–1.68 eV), thereby reducing the Br content required for current matching. Since the severity of photo-induced phase segregation increases with increasing Br content and becomes particularly pronounced for bandgaps above approximately 1.70 eV, CIGS/perovskite tandems generally experience a lower intrinsic driving force for phase segregation than Si/perovskite or all-perovskite tandems. Nevertheless, defect formation, ion migration, and interfacial recombination remain critical factors governing device performance and long-term operational stability. Therefore, a comprehensive understanding of defect formation mechanisms and effective defect-engineering strategies remains essential for realizing high-efficiency and durable CIGS/perovskite tandem solar cells [110].

### Why wide-bandgap (1.63–1.8 eV) perovskites are more defect-prone?

The compositional and structural modifications that are critical to achieving bandgap widening cause inherent defects in WBG perovskites. Notably, increasing the bromine (Br) ion ratio in the perovskite absorber intrinsically widens the bandgap of the perovskite absorber [111]. The deeper-lying Br 4p orbitals aid in shifting the valence band maximum (VBM) to lower levels [112]. Thus, as the Br fraction increases in mixed halide perovskites, the bandgap can be tuned from ~ 1.5 to 1.8 eV. However, increasing Br ion incorporation significantly modifies defect energetics by lowering the formation energy of halide vacancies (V_Br_), thereby increasing defect density [113]. In addition, Br-rich compositions introduce lattice strain and reduce dielectric screening, thereby diminishing the intrinsic defect tolerance of the perovskite lattice and forming deep-level traps [114]. These deep traps act as non-radiative recombination centers, increasing trap-assisted recombination, limiting carrier lifetimes and diffusion lengths. Moreover, the rapid crystallization kinetics associated with Br-rich precursor systems typically yield smaller grain sizes and a higher density of GBs, which serve as sites for defect accumulation and ion migration [115, 116]. Collectively, these effects fundamentally induce higher defect formation in WBG perovskites than narrow bandgap counterparts, causing a limitation to their photovoltaic performance.

Photo-induced halide segregation represents an important instability mechanism in mixed-halide WBG perovskites, although its severity strongly increases with Br content and is therefore considerably more pronounced in higher-bandgap (> ~1.70 eV) compositions than in the ~ 1.63–1.68 eV absorbers typically relevant to CIGS/perovskite tandems [117]. Under illumination or applied bias, mobile halide ions (Br and I) redistribute within the lattice, driving phase segregation, creating Br-rich (wide-bandgap) and I-rich (narrow-bandgap) domains [118, 119]. The formation of these iodide-rich regions introduces localized bandgap inhomogeneity and creates energetically favorable recombination centers that direct charge carriers into low-bandgap domains, thereby increasing non-radiative recombination pathways. This dynamic segregation process not only degrades spectral stability but also contributes directly to V_OC_ losses [120, 121]. Thus, compared to ~ 1.5 eV narrow bandgap counterparts, the WBG perovskites exhibit larger V_OC_ deficits reflecting their inability to suppress non-radiative recombination. Fundamentally, the origin of V_OC_ loss in WBG perovskites can be linked to a combination of bulk and interface defects, deep trap states, and recombination at GBs and segregated phases. The resulting reduction in quasi-Fermi level splitting, driven by increased trap-assisted recombination and defect-mediated carrier losses, ultimately limits the achievable V_OC_ [122]. In this context, the reduced defect tolerance of WBG perovskites arising from their altered electronic states and increased ionic mobility necessitates targeted defect mitigation strategies. Advanced approaches, including compositional engineering, defect passivation, and suppression of ion migration, are required to approach the radiative efficiency limits required for high-performance tandem devices.

### Intrinsic defect types in wide-band gap perovskites

The defects in the WBG perovskites are inherently dominated by point defects, halide segregation, and GB imperfections. These defects can be intrinsically traced to the increased Br ion ratio in the WBG perovskites that results in increased trap densities, enhanced ion migration, and inferior electronic properties.

- Point defects

**Fig. 6:**
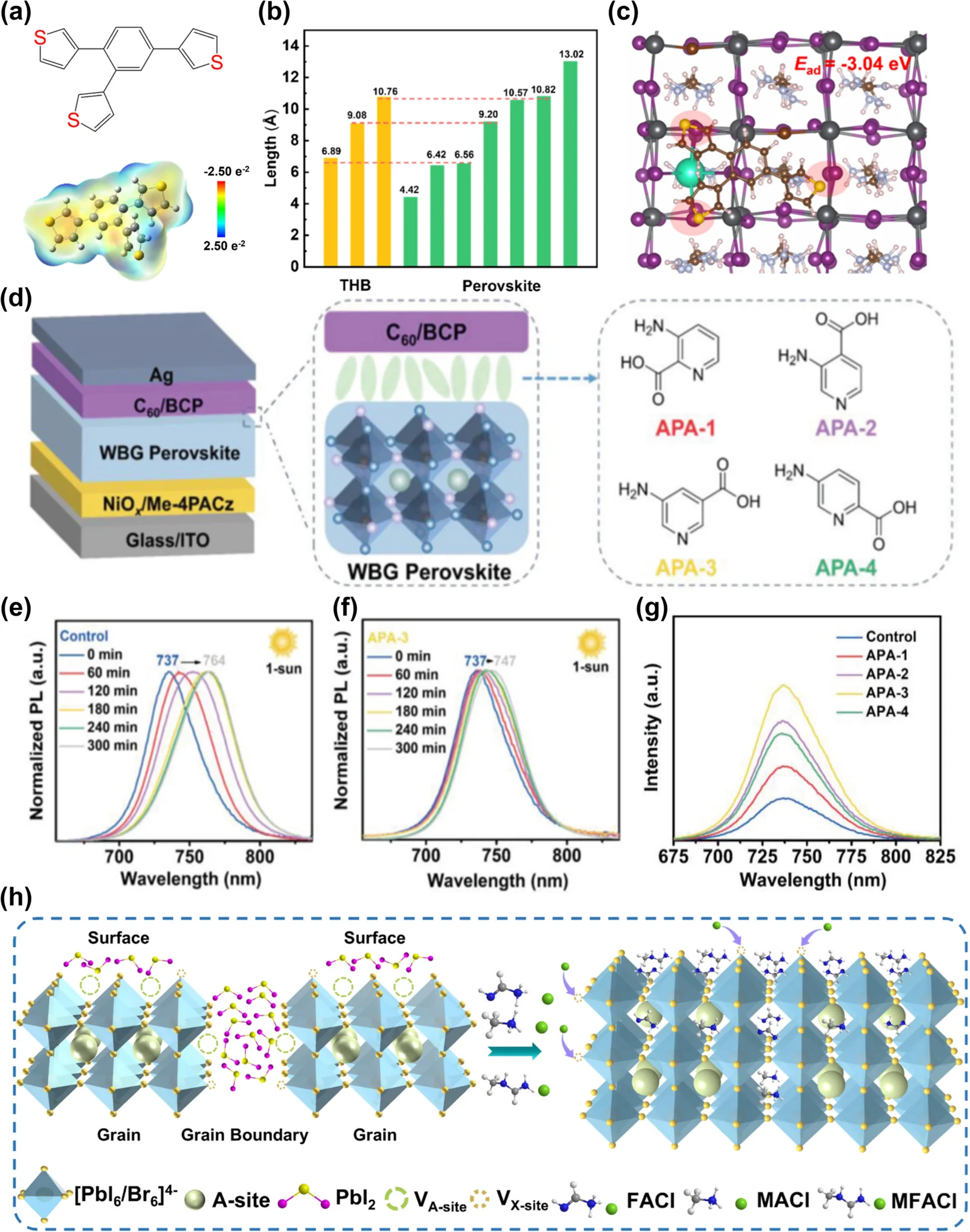
**a** Chemical structure of the THB molecule and its calculated ESP. **b** Summary of distances between adjacent X in perovskites and S atoms in THB molecules. **c** Theoretical models of perovskites with passivation of uncoordinated Pb^2+^ by THB. Reproduced with permission from Ref. [124]. **d** The configuration of WBG PSCs and the chemical structure of the pyridine derivatives. **e**–**f** PL spectra of control and APA-3 modified WBG PSCs illustrating the reduction in halide segregation. **g** Steady-state PL spectra of the control, APA-1, APA-2, APA-3, and APA-4 treated perovskite films. Reproduced with permission from Ref. [132]. **h** Schematic diagram of perovskite GB passivation mechanism. Reproduced with permission from Ref. [138]

The dominant intrinsic defects in the WBG perovskites are halide vacancies (V_I_, V_Br_), Pb-related defects (V_Pb_, Pb_i_), and antisite defects (I_Br_, Br_I_) [123–125]. In the Br-rich WBG perovskite, the defect formation energies are strongly governed by halide chemical potential, Fermi-level position, and local stoichiometry. First-principles DFT calculations have shown that V_Br_ can exhibit relatively low formation energies, approximately 0.4–1.2 eV under specific Br-poor/Pb-rich conditions, and generally forms shallow donor states [126]. While isolated V_Br_ defects typically form shallow donor levels, their relatively low formation energy triggers an increased equilibrium concentration of mobile halide vacancies that accelerates ionic migration pathways under operational bias [126, 127]. This alteration in the defect kinetics can be assigned to the stronger Pb-Br bonding and associated lattice strain, which reorganizes local structural relaxation. Although isolated V_Br_ defects are generally electronically shallow, their high mobility promotes the formation of defect complexes and halide segregation, thereby indirectly enhancing non-radiative recombination. In parallel, antisite defects and V_Pb_ further disrupt local charge neutrality, promote trap formation, and increase recombination losses [123, 128]. DFT models reveal that the stability and type of antisite defect dictating this degradation are highly dependent on the local chemical potential. Specifically, iodine-on-bromine antisites (I_Br_) possess very low formation energies (frequently below 0.5 eV under iodine-rich growth regimes) and may create discrete, deep-level hole traps near the mid-gap [126, 129]. Conversely, the energetic barriers for lead-related defects shift dramatically with local stoichiometry; under Pb-rich conditions, the formation energies of harmful lead interstitials Pb_i_ and lead-on-halide antisites Pb_X_ drop to 0.3–0.8 eV, creating high-capture-cross-section electron traps. In contrast, lead vacancies V_Pb_ function as harmless shallow acceptors with high formation energies under halide-rich conditions, rendering them electronically benign. As a result, the elevated density and activity of point defects in WBG perovskites suppress quasi-Fermi level splitting, increase V_OC_ deficits, and render performance sensitive to processing conditions. Among these competing species, first-principles analyses clarify that the mid-gap I_Br_ antisite, halide interstitials (I_i_, Br_i_), and lead interstitials (Pb_i_) are among the dominant contributors to the observed V_OC_ deficit due to their deep energy levels and high nonradiative recombination rates [130]. Furthermore, the highly mobile V_Br_ vacancies facilitate ion migration under illumination, which drives local phase segregation into iodide-rich domains. This forms severe energetic sinks that funnel and trap photogenerated carriers, fundamentally limiting the achievable quasi-Fermi level splitting of the wide-bandgap top cell. For instance, 1,2,4-tris(3-thienyl)benzene (THB) molecules have been introduced onto the perovskite surface to counteract the detrimental effects associated with halogen ion vacancies (Fig. 6a). The sulfur (S) atoms present in the THB molecule exhibit strong chemical affinity toward undercoordinated Pb^2+^ centers on the perovskite surface, enabling effective defect passivation. In addition, the THB molecules can occupy the migration channels associated with halide ions, thereby inhibiting ion migration and improving the structural stability of the perovskite lattice. The electrostatic potential (ESP) distribution of THB further reveals a highly concentrated negative charge density around the sulfur atoms due to their strong electronegativity, which facilitates strong electrostatic interaction with positively charged Pb^2+^ defect sites. To better understand the geometric compatibility between THB and the perovskite lattice, a Cs_0.05_(FA_0.77_MA_0.23_)_0.95_Pb(I_0.7_Br_0.3_)_3_ structure was constructed and compared with the molecular configuration of THB (Fig. 6b). Interestingly, the spacing between the three sulfur atoms in THB closely matches the typical halogen vacancy spacing on the perovskite surface, enabling simultaneous coordination of multiple undercoordinated Pb^2+^ sites. The corresponding passivation mechanism of THB on the surface of Cs_0.05_(FA_0.77_MA_0.23_)_0.95_Pb(I_0.7_Br_0.3_)_3_ films is illustrated in Fig. 6c. Moreover, density functional theory (DFT) calculations demonstrated a strong adsorption energy of − 3.04 eV between the sulfur atoms of THB and Pb^2+^ sites on the perovskite surface [124].

(b) Halide segregation

Photo-induced halide segregation represents an important composition-dependent instability in mixed-halide WBG perovskites, particularly at higher Br contents [125]. The initially homogeneous lattice upon illumination or bias forms I-rich (narrow bandgap) and Br-rich (wide bandgap) domains. The formation of I-rich domains creates local bandgap minima, which act as sinks for charge carriers and effectively create easy pathways for electrons and holes, enhancing non-radiative recombination. Additionally, the I-rich domains stabilize deep defect levels, resulting in the coexistence of shallow and deep defect levels [131]. The dominating deep-level defects regulate the recombination dynamics and increase the V_OC_ deficit. Further, the reversible nature of the halide segregation (known as Hoke’s Law) leads to localized bandgap fluctuations, creating instability in the spectral response [125]. Thus, halide segregation in WBG perovskites leads to suboptimal performance, increased hysteresis, and compromised long-term stability. Recently, a series of pyridine-derivative isomers (Fig. 6d**)** of 3-amino-2-pyridinecarboxylic acid (APA-1), 3-amino-4-pyridinecarboxylic acid (APA-2), 5-amino-3-pyridinecarboxylic acid (APA-3), and 5-amino-2-pyridinecarboxylic acid (APA-4) have shown effectiveness in suppressing phase segregation and migration in WBG perovskites. Owing to the presence of identical functional groups, including amino groups, carboxyl groups, and pyridine N-terminals, these molecules can effectively interact with undercoordinated Pb^2+^ ions and halide vacancies, thereby reducing defect density and suppressing ion-migration pathways. Photoluminescence (PL) measurements revealed that the untreated control perovskites exhibited a pronounced red shift under continuous illumination, indicating severe halide segregation and the formation of I-rich domains (Fig. 6e). In contrast, the APA-3-treated films displayed significantly smaller PL shifts, confirming effective suppression of light-induced phase segregation (Fig. 6f). Moreover, the APA-3-treated devices exhibited the highest steady-state PL intensity, suggesting lower defect density (Fig. 6g). These results highlight the effectiveness of multifunctional molecular passivation in suppressing light-induced phase segregation in WBG perovskites [132].

(c) Grain boundary defects

The polycrystalline WBG perovskites constitute GBs that are defect hotspots, arising due to their structural disorder and high density of under-coordinated species [133]. These regions typically comprise undercoordinated Pb^2+^ centers, organic cation vacancies, and a large concentration of halide vacancies [134, 135]. GBs are the preferential pathways for ion migration, enabling the accumulation of halide ions and vacancies under illumination or applied bias. This ionic restructuring creates local electric fields, accelerating further defect generation. The GB-mediated ion migration is a primary origin of J–V hysteresis, as the dynamic movement of ions modulates the internal electric field and alters charge extraction [136, 137]. During continuous operation under illumination and thermal stress, these coupled ionic-electronic processes determine structural degradation and phase instability. Thus, apart from increasing non-radiative recombination and *V*_OC_ deficit, the GB defects limit the operational stability of WBG perovskites. To address these issues, various GB passivation strategies have been developed. Figure 6h schematically illustrates the mitigation of GB defects using N-methylformamidinium (MFA^+^) chloride, formed through the cation-exchange reaction between formamidinium hydrochloride (FACl) and methylammonium hydrochloride (MACl). The incorporation of MFA^+^ species effectively passivates defect-rich GB regions, suppresses ion migration pathways, and enhances the structural stability of WBG perovskite films under operational conditions [138]**.**

### Compositional engineering strategies

Compositional tuning is a versatile approach to mitigate defect formation in the WBG perovskites. By fine-tuning the halide composition, A-site cations, and precursor chemistry, the perovskite lattice can be stabilized to suppress ion migration and reduce defect density.

- Mixed cation engineering

Mixed-cation engineering, involving combinations of formamidinium (FA^+^), cesium (Cs^+^), and methylammonium (MA^+^), has proven highly effective in stabilizing WBG perovskite structures and suppressing defect formation. Incorporation of multiple A-site cations (considering the Goldschmidt tolerance factor), namely, FA^+^, Cs^+^, and MA^+^_,_ is known to suppress undesired phase transitions and reduce lattice strain, yielding a thermodynamically stable perovskite [139, 140]. This technique is predominantly used in large lattice-distortion Br-rich WBG perovskites. The Cs^+^ and FA^+^ are incorporated to improve structural robustness and thermal stability, and carrier transport, respectively. Further structural refinement through the inclusion of the alkali metal cations, such as Rb^+^ and K^+^, assists additional defect passivation and film morphology improvement [140–142]. These smaller cations occupy the interstitial positions or segregate to GBs, suppressing non-radiative recombination. Mechanistically, the mixed-cation perovskites possess high activation energy for ion migration, which mitigates halide segregation under illumination or electrical bias [143]. Simultaneously, these smaller cations stabilize the local lattice environment, suppress the formation of deep-level defects, enhance the quasi-Fermi level splitting, and reduce V_OC_ losses.

(b) Halide composition optimization

Precise control of halide composition is critical in balancing bandgap tuning with structural and phase stability in WBG perovskites. Increasing the Br ion ratio is essential to achieve band gaps in the 1.65–1.8 eV range; however, introducing excess Br can promote phase segregation and defect formation. Therefore, identifying an optimal Br/I ion ratio is key to forming compositionally homogeneous WBG absorbers while mitigating ionic instability [108, 144]. Studies establish an optimally balanced and narrow compositional window associated with band gaps of ~ 1.68–1.72 eV, where the thermodynamic driving force for halide segregation is reduced. Additionally, these balanced halide compositions suppress the formation of deep-level traps. Further, controlling the halide distribution across the bulk and surface regions improves bandgap homogeneity and restricts carrier funneling into I-rich low bandgap domains.

(c) Additive engineering

Additive engineering has emerged as a strategy for simultaneously regulating crystallization dynamics and passivating defects in WBG perovskite films. Additives such as methylammonium chloride (MACl) and ammonium chloride (NH_4_Cl) are widely employed to control nucleation and grain growth, promoting the formation of larger grains with reduced GB density [145–148]. These chloride-based species can transiently incorporate into the perovskite lattice during film formation and subsequently volatilize upon annealing, yielding a more ordered crystal structure with diminished defect density. Apart from crystallization, alkali metal salts assist in defect passivation by interacting with halide vacancies and undercoordinated ions [149]. Similarly, Lewis base additives, such as amines, thiols, carbonyls, etc., coordinated with undercoordinated Pb^2+^, passivate both surface and GB defects [150, 151]. Other smaller molecules are known to stabilize the lattice through ionic interaction or hydrogen bonding, limiting the lattice distortions [152, 153]. Apart from defect healing and suppressing the deep-level trap states, additive incorporation refines the crystallization kinetics through controlled nucleation, producing films with uniform grain growth.

### Surface passivation strategies

In WBG perovskites, surface and near-surface defects disproportionately govern the device losses. Thus, surface passivation represents a critical strategy for suppressing defect-induced non-radiative recombination in WBG perovskites. The higher Br ion ratio and associated structural disorder in WBG perovskites give rise to an increased density of undercoordinated ions and vacancy defects at the surface and GBs. These sites act as recombination centers and assist ion migration, causing V_OC_ deficit, instability, and hysteresis. Hence, effective surface passivation targets the chemical neutralization of defect states through coordinated bonding and ionic compensation. Additionally, the passivation layers improve band alignment and modulate interface properties to minimize charge accumulation at defective interfaces [154].

- Molecular passivation

Molecular passivation utilizes functional molecules such as Lewis bases (thiols, amines, pyridine derivatives), phosphonic acids, and carboxylic acids to selectively interact with under-coordinated Pb^2+^ ions present at the perovskite surface and GBs. The electron-donating functional groups (–NH_2_, –SH, –COOH, –PO_3_H_2_) present in these molecules form coordinated bonds with Pb^2+^ ions to saturate the dangling bonds and eliminate deep trap states [155, 156]. This coordination-driven passivation effectively reduces the density of the electronically active defect states to suppress non-radiative recombination. Beyond defect neutralization, molecular passivation also modulates interface energetics by tuning local band alignment with adjacent charge transport layers, facilitating more efficient charge extraction and reducing interface recombination losses. The consequent reduction in surface trap density enhances quasi-Fermi level splitting and promotes radiative recombination, directly contributing to reduced *V*_OC_ deficits. Additionally, these molecular layers can serve as protective barriers against environmental factors such as moisture and oxygen, further improving device stability [157]. Therefore, molecular passivation is effective in addressing surface defects and enhancing both efficiency and stability in WBG perovskites.

(b) Ionic passivation

In ionic passivation, the halide and pseudo-halide salts are incorporated to compensate for the halide vacancies and neutralize charged defect sites within the perovskite lattice and at interfaces [158]. The local stoichiometry is restored by the supply of halide ions (I^–^ or Br^–^) that occupy the vacancy sites (V_I_, V_Br_) to reduce defect density. Pseudo-halide ions, such as thiocyanate (SCN^–^), tetrafluoroborate (BF_4_^–^), and formate, have emerged as active passivating agents due to their strong coordination affinity with Pb^2+^ and their ability to stabilize the lattice without distorting the crystal structure [159, 160]. These species can occupy defect sites or form stable complexes with under-coordinated ions, effectively eliminating deep-level trap states and increasing the activation energy for ion migration. Importantly, ionic passivation also influences interface energetics by reducing band offsets at critical interfaces, which improves charge separation and extraction [161–163]. The combined reduction in trap density and improved band alignment enhances radiative efficiency, leading to higher quasi-Fermi level splitting and reduced V_OC_ deficits. Additionally, by stabilizing mobile ionic species and limiting their redistribution under operational bias, ionic passivation mitigates hysteresis and enhances long-term device stability [164]. Consequently, ionic passivation is a key enabler for achieving high-efficiency and durable WBG perovskite solar cells.

(c) 2D/3D perovskite capping

An advanced and effective surface passivation strategy mainly uses Ruddlesden-Popper (RP) phases to form the 2D/3D perovskite heterostructures. Here, a bulky organic cation is used to form an ultrathin 2D perovskite layer on the surface of the 3D WBG perovskites, creating a graded interface with distinct structural and electronic properties [109, 165]. The 2D capping layer modifies the surface energy of the WBG perovskites by passivating the defects and reducing the undercoordinated ions and halide vacancies on the surface. Critically, the resulting 2D/3D interface often forms a cascaded band alignment to facilitate charge extraction [166]. This frequently leads to suppressed recombination velocity and improves quasi-Fermi level splitting. Additionally, the 2D capping layer physically blocks the ion migration pathways by increasing the activation barrier to reduce hysteresis. The hydrophobic nature of the many 2D perovskite capping layers also enhances the stability by shielding the underlying 3D WBG perovskite layers [166]. In total, the 2D/3D passivation simultaneously assists in defect passivation, band alignment, and stability improvement in WBG perovskites.

### Suppression of ion migration

Suppressing ion migration is critical for WBG perovskites, especially in tandem architecture where prolonged illumination, elevated temperatures, and built-in electric fields accelerate ionic movement and exacerbate device degradation. The Br-rich WBG perovskites comprise mobile ionic species, such as the halide ions (I^–^, Br^–^), vacancies (V_I_, V_Br_), and cation-related defects. Under applied bias, these species experience field-driven redistribution, inducing dynamic fluctuations in the electric fields, band bending, and interface properties. These effects collectively emerge as pronounced hysteresis, reduced FF, and leading to V_OC_ losses. A primary strategy to weaken ion migration includes GB blocking [167, 168]. In the GB blocking mechanism, passivation agents or secondary phases are introduced to both physically and chemically stabilize the GBs. By eliminating defect-rich diffusion pathways and passivating under-coordinated ions, GB blocking increases the activation energy for ion transport and suppresses ionic flux under operational conditions. In parallel, the incorporation of interface dipole layers, such as self-assembled monolayers (SAMs), fullerene derivatives, or tailored molecular interlayers, enables precise control of local electric fields at critical interfaces (perovskite/ETL and perovskite/hole transport layer (HTL)) [169]. These dipolar layers homogenize the distribution of the internal electric field to minimize ion drift and interface degradation. Other approaches focus on exploiting compositional engineering and defect passivation to reduce the overall density of the mobile ionic species in the WBG perovskites. The introduction of mixed cations, pseudo-halides, alkali metals, etc., stabilizes the lattice structure by suppressing vacancy formation to limit the ionic mobility at its origin [170–172]. Together, these strategies restrict ion migration, stabilize band alignment, and suppress hysteresis. For CIGS/perovskite tandem solar cells where the top WBG perovskite must operate reliably under continuous illumination and electrical bias, controlling ion migration is essential for maintaining spectral stability, minimizing interface recombination, and ensuring long-term operational durability. Consequently, effective suppression of ion migration directly translates into improved device stability, sustained V_OC_, and enhanced overall performance.

### Perovskite/charge transport layer interface engineering

The performance and stability of single-junction perovskite solar cells and CIGS/perovskite tandem architectures are governed by the interface properties between the perovskite absorber and the adjacent charge-transport layers. In WBG perovskites, where defect densities and ionic mobility are inherently higher, interface recombination losses become a dominant limitation in achieving high V_OC_ and FF. Unoptimized interfaces degrade charge extraction and accelerate device degradation by introducing traps, chemical instability, and band misalignment at the critical interface [173]. Therefore, precise control of interface chemistry, energetics, and defect states is essential for minimizing non-radiative recombination and enabling stable and high-efficiency tandem operation.

#### Perovskite/ETL interfaces

The interface between the perovskite absorber and ETL plays a critical role in charge extraction, recombination dynamics, and long-term stability. In tandem architecture, widely employed ETLs include SnO_2_, TiO_2_, ZnO, atomic layer deposition (ALD)-based oxides (e.g., SnO_2-x_, ZnSnO_x_, TiO_2-x_), and organic ETLs such as fullerene derivatives. While these materials offer favorable band alignment and electron selectivity, their surfaces inherently constitute intrinsic defects that act as recombination centers and trigger interface degradation.

A primary source of interface defects in metal-oxide ETLs is the presence of oxygen vacancies, which introduce shallow donor states but can also function as electron traps under certain conditions [174, 175]. In SnO_2_ and TiO_2_, a high density of oxygen vacancies near the interface increases carrier scattering and facilitates non-radiative recombination, limiting charge extraction efficiency and reducing *V*_OC_. In addition, hydroxyl (–OH) groups, primarily formed during solution processing or ambient exposure, introduce polar surface states that can trap charges and assist undesirable chemical interactions with the perovskite layer [176–178]. These hydroxyl-rich surfaces can also accelerate perovskite decomposition by promoting deprotonation of organic cations. Notably, in ZnO ETL, the presence of surface hydroxyl groups is chemically reactive with hybrid perovskites, limiting their long-term stability [179]. Additionally, the surface traps, arising from the under-coordinated metal ions and dangling bonds, further exacerbate trap-assisted recombination at the perovskite/ETL interface. These interface defects reduce quasi-Fermi level splitting and lead to V_OC_ deficit. Moreover, chemical reactions at the perovskite/ETL interface, such as ion exchange or interface redox reactions, can lead to the formation of a defective interface, further degrading the electronic quality and stability of the devices [180, 181]. In WBG perovskites, where ionic mobility is high, migrating halide ions can accumulate at the ETL interface. The interaction of these mobile species with pre-existing defect sites promotes the formation of additional trap states and accelerates interface degradation under operational conditions. To address these interface challenges, a range of mitigation strategies has been developed to passivate defects, stabilize surface chemistry, and optimize energy level alignment. Among these, surface treatments such as UV-ozone and plasma exposure are widely employed to remove residual organic contaminants and reduce hydroxyl group density on ETLs. These treatments also enhance surface wettability, enabling more uniform perovskite film formation and improved interface contact. Nevertheless, the effectiveness of such treatments is highly sensitive to processing conditions. Excessive treatment can introduce additional defects or damage the ETL surface.

SAMs have been pioneered as an effective technique, as it offers precise control over surface chemistry and electronic properties. SAM molecules passivate surface defects by chemically anchoring to under-coordinated sites and introduce dipole moments to tune the work function of the ETLs [182, 183]. This enables optimized band alignment at the perovskite/ETL interface, enabling efficient electron extraction. Importantly, the SAMs act as a barrier layer, minimizing the direct chemical interaction between the perovskite absorber and ETL, which aids in stability improvement [184].

ALD-grown conformal interlayers provide a direct route for interface engineering in tandem perovskite devices. Owing to its self-limiting surface reactions, ALD enables angstrom-level thickness control and exceptional conformality over complex and rough surfaces, allowing precise modulation of interface properties. Ultrathin ALD layers, such as SnO_2_, Al_2_O_3_, ZrO_2_, etc., can efficiently passivate surface defects, suppress oxygen vacancies, and establish chemically robust interfaces with the perovskite absorber [185, 186]. Moreover, ALD-derived passivation offers tunable electronic properties by reducing band offsets at the perovskite/ETL interface [187]. The conformal nature of the ALD-grown passivation also ensures uniform coverage across GBs and nanoscale roughness, which becomes critical for large-area tandem architectures.

Collectively, these interface strategies ensure reduced interface recombination by eliminating trap states and suppressing defect-assisted carrier capture at the ETL/perovskite interface. By enabling precise control over band alignment, they facilitate efficient charge extraction. Simultaneously, these approaches inhibit ion accumulation and interface degradation, thereby reducing hysteresis and enhancing operational stability. Overall, the perovskite/ETL interface is a critical component in tandem solar cells, and its optimization requires a synergistic combination of surface passivation, chemical stabilization, and energy level engineering.

Recent experimental studies and drift–diffusion device simulations have established a direct quantitative relationship between interface defect density and initial photovoltaic performance [188]. Numerical modeling indicates that increasing the interface defect density (N_it_​) from approximately 10^10^ to 10^12^ cm^−2^ substantially enhances Shockley–Read–Hall (SRH) recombination at the perovskite/ETL interface, whereas maintaining N_it_​ below approximately 10^10^ to 10^11^ cm^−2^ minimizes interface recombination and preserves efficient charge extraction [189]. Experimental visualization of interface recombination further demonstrated that minority-carrier recombination at each transport-layer interface introduces an additional free-energy loss of approximately 80 meV, which significantly limits the achievable V_OC_​. The introduction of ultrathin passivation interlayers effectively suppresses these interface recombination losses, leading to higher quasi-Fermi level splitting and improved device performance [188]. These findings demonstrate that interface defect density and the associated interface recombination losses are critical quantitative descriptors governing the electronic quality of the perovskite/ETL interface.

#### Perovskite/HTL interfaces

The perovskite/HTL interface is particularly important in CIGS/perovskite tandems because the perovskite top cell is generally implemented in an inverted (p–i–n) configuration. Consequently, buried hole-selective contacts based on NiO_x_, phosphonic-acid SAMs, and NiO_x_/SAM bilayers are substantially more relevant to this tandem architecture than conventional doped Spiro-OMeTAD layers predominantly employed in n–i–p single-junction devices [190, 191]. The tandem-relevant HTL must simultaneously provide favorable valence-band alignment, efficient hole selectivity, low interfacial recombination, chemical compatibility with the subsequently deposited perovskite layer, and sufficiently uniform coverage over the underlying CIGS-derived interconnection surface. Among inorganic hole-selective contacts, NiO_x_ is particularly attractive for inverted perovskite and tandem architectures because of its wide optical bandgap, relatively high transparency, thermal robustness, and compatibility with low-temperature processing. Nevertheless, the electronic properties of the NiO_x_/perovskite interface are highly sensitive to its surface chemistry. Ni vacancies, oxygen-related defects, variations in Ni^2+^/Ni^3+^ oxidation states, and surface hydroxyl species can modify the NiO_x_ work function and introduce electronically active states at the buried interface [192–194]. These surface and bulk defects can induce non-radiative recombination, unfavorable interfacial band bending, and local charge accumulation. Moreover, ionic redistribution at the NiO_x_/perovskite interface can further alter the interfacial electric field during operation, contributing to hysteresis and long-term instability [195]. Therefore, controlling the defect chemistry and surface termination of NiO_x_ is essential for obtaining reproducible and low-loss hole-selective contacts.

SAM-based hole-selective contacts have consequently attracted substantial attention for inverted perovskite solar cells because their molecular-scale thickness minimizes parasitic optical absorption while enabling simultaneous control of surface energetics and chemical passivation [196]. Phosphonic-acid-based SAMs can chemically anchor to oxide surfaces and tune the substrate work function through their molecular dipole, thereby improving energetic alignment with the perovskite valence band [197]. However, the performance of SAM-based hole-selective contacts depends strongly on molecular surface coverage, orientation, anchoring density, and wettability during subsequent perovskite deposition. Incomplete or spatially non-uniform SAM coverage can expose the underlying oxide and generate local recombination-active regions, whereas excessive molecular aggregation may impede charge transfer. These limitations become particularly important for CIGS/perovskite tandems because the buried hole-selective interface is formed on an interconnection layer that can retain appreciable nanoscale roughness and chemical heterogeneity. Accordingly, achieving homogeneous molecular coverage over a non-ideal tandem substrate remains an important integration challenge.

Hybrid NiO_x_/SAM hole-selective contacts provide a particularly promising route to overcome these limitations. In this configuration, NiO_x_ provides a continuous and mechanically robust hole-selective layer, while the SAM modifies the NiO_x_ surface, passivates electronically active sites, and tunes the interfacial work function [198]. Such complementary interface engineering can reduce non-radiative recombination and improve hole extraction while retaining the processing robustness required for tandem integration. The relevance of this approach is also reflected in recent high-efficiency CIGS/perovskite tandem architectures, where NiO_x_, carbazole-phosphonic-acid SAMs, or their combinations have been employed at the buried hole-selective interface [13]. Consequently, optimization of the NiO_x_/SAM/perovskite junction should be considered a tandem-specific interface-engineering problem involving not only energy-level alignment but also surface coverage, chemical stability, and defect passivation.

Additional molecular or ultrathin interfacial modifiers can further reduce recombination at the buried hole-selective contact by passivating electronically active sites on the HTL surface, modifying surface energy, and promoting uniform perovskite nucleation. Functional molecules can also tune the interfacial dipole and thereby improve energetic alignment between the hole-selective contact and the perovskite absorber [196, 199–202]. However, such interlayers must remain sufficiently thin and electronically compatible to avoid introducing additional transport resistance. Effective buried-interface engineering therefore requires simultaneous optimization of chemical defect passivation, surface energetics, perovskite growth, and efficient hole transfer. In this context, molecularly controlled interfaces are advantageous because defect passivation, surface-energy modification, and interfacial dipole formation can be combined within an ultrathin contact [196].

Beyond determining the initial photovoltaic performance, defects at the buried hole-selective interface can also influence ionic redistribution and the stability of mixed-halide WBG perovskites. Using MAPb(Br_0.5_I_0.5_)_3_ as a model mixed-halide system, Knight et al. demonstrated a direct relationship between the extent of photo-induced phase segregation and the fraction of carriers undergoing trap-mediated recombination, estimating that reducing the trap-related recombination rate to ~< 10^5^ s^−1^ (corresponding to a photoluminescence lifetime of >5 μs) would be required to sufficiently suppress phase segregation under device-relevant conditions [203]. Furthermore, first-principles calculations by Meggiolaro et al. showed that defect formation is energetically favored at perovskite surfaces and GBs relative to the bulk, providing a mechanistic basis for enhanced ionic migration through these regions [204]. These studies collectively indicate that reducing the density of electronically and ionically active defects at buried interfaces can simultaneously mitigate non-radiative recombination and reduce pathways that facilitate ionic redistribution.

For monolithic CIGS/perovskite tandems, the principal challenge is therefore not the identification of a generic HTL, but the realization of a buried hole-selective interface that remains electronically selective and chemically stable when integrated onto the morphologically and chemically heterogeneous CIGS-derived interconnection stack. NiO_x_, phosphonic-acid SAMs, and particularly NiO_x_/SAM hybrid contacts provide promising routes because they combine robust surface coverage with molecular-scale control of defect passivation and energy-level alignment. Their optimization must therefore consider not only intrinsic charge-selective properties but also conformality, interfacial recombination, perovskite nucleation, and compatibility with tandem processing.

#### Interface dipole engineering

Interface dipole engineering has been a versatile strategy for optimizing interface energetics in WBG perovskite-based tandem solar cells. Unlike conventional passivation approaches that primarily focus on reducing defect density, interface dipole engineering mainly targets the modulation of interface energetics through vacuum level shifts and work function tuning at the perovskite/charge transport layer interface. Nevertheless, many dipolar interface molecules simultaneously contribute to chemical defect passivation by coordinating with undercoordinated surface species, thereby enabling concurrent energetic optimization and recombination suppression. The incorporation of an interface dipole moment shifts the local vacuum level and effectively modifies the work function of adjacent layers [205–207]. This results in a controlled alignment of the perovskite conduction and valence bands with the transport layers, improving charge extraction. For perovskite/ETL interfaces, dipole layers can reduce unfavorable conduction band offsets (i.e., converting “cliff-like” alignments into optimal flat or slightly “spike-like” configurations), while for hole-selective interfaces, they can optimize valence band alignment to facilitate efficient hole transfer [208, 209]. As a result, the carrier accumulation at interfaces is reduced, suppressing trap-assisted recombination. The net effect is a reduction in carrier accumulation at interfaces, which otherwise promotes trap-assisted recombination. Additionally, the enhancement of the built-in electric field across the junction improves charge separation and directional carrier transport, further limiting recombination losses [210].

**Fig. 7:**
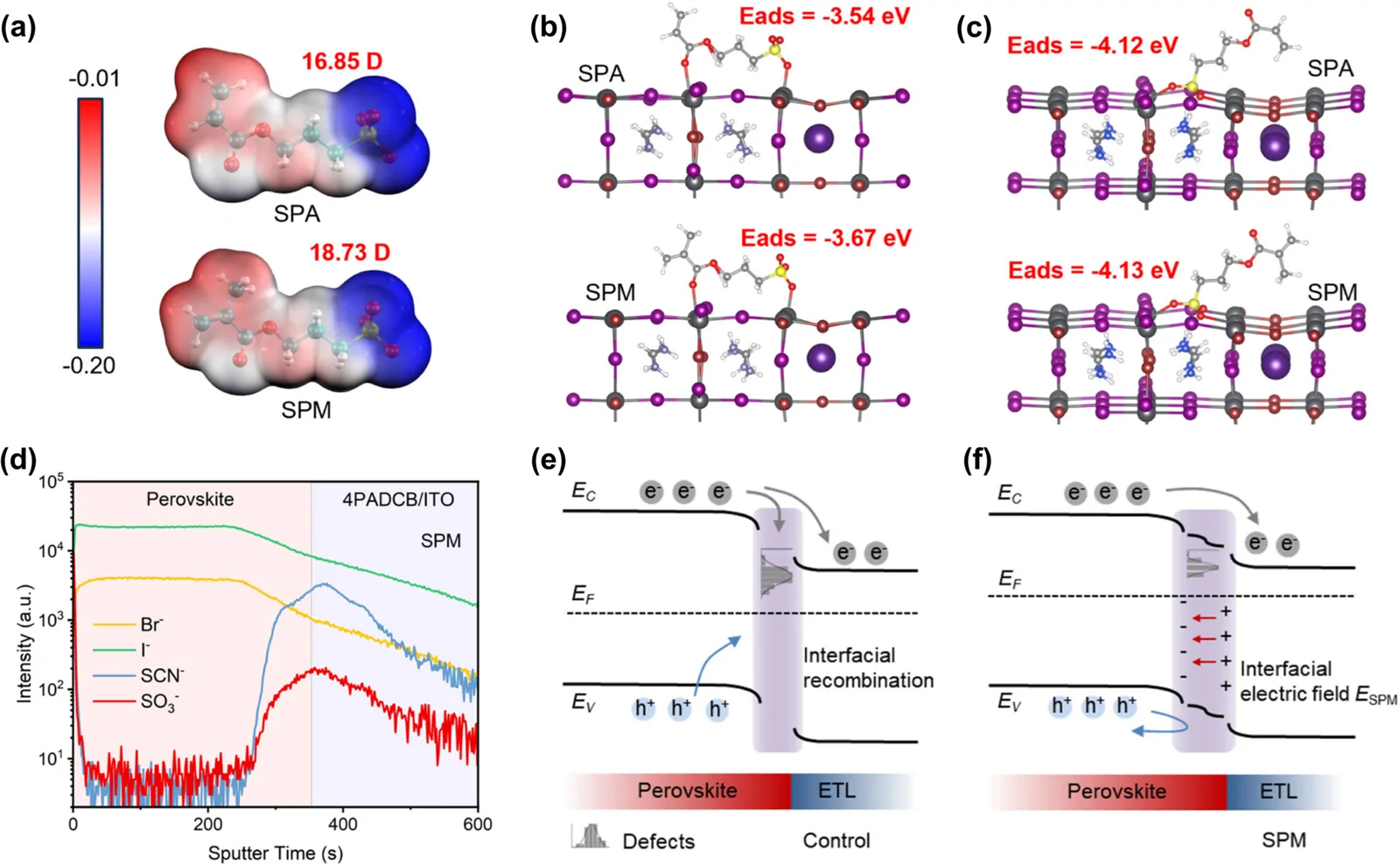
**a** Structure and ESP of SPA and SPM ligands and molecular dipole moment. The different adsorption configurations: **b** parallel orientation and **c** vertical orientation on the perovskite surface with different anchoring agents. **d** ToF–SIMS profile of the SPM-treated perovskite film. **e** and **f** Interface energy level diagram with the preferred dipole moment and carrier transport. Reproduced with permission from Ref. [221]

Owing to their ability to form highly ordered and ultrathin films with well-defined molecular orientation, SAMs are the most used materials for interface dipole engineering. Typically, SAMs comprise anchoring groups (i.e., carboxylic acids, phosphonic acids) that bind to the oxide surfaces and functional tail groups that introduce a permanent dipole [211]. By judicious selection of molecular structure and dipole orientation, SAMs enable precise tuning of the work function of charge transport layers, thereby optimizing band alignment with the perovskite absorber and minimizing interface energy barriers [212, 213]. In addition to a reduction in band offsets, SAMs reduce the trap density by assisting in passivating the surface defects by chemically interacting with undercoordinated sites. This dual functionality showcases interface dipole engineering with SAMs as a convenient technique for improving both V_OC_ and FF in WBG perovskite devices. Likewise, fullerene derivatives, widely used as ETLs or interface modifiers, also contribute significantly to dipole engineering. Molecules such as PCBM and its functionalized variants can form interface layers that not only passivate defects but also introduce interface dipoles due to their electron-withdrawing nature [214, 215]. These dipoles support favorable downward band bending at the perovskite/ETL interface by suppressing back recombination. Furthermore, fullerene-based layers can act as a barrier against ion migration and interface chemical reactions, improving stability [216]. Their compatibility with solution processing and ability to conformally coat rough perovskite surfaces make them attractive for large-area and tandem devices. Also, phosphonic acid-based molecules represent another important class of dipole-inducing materials, particularly for oxide-based charge transport layers such as SnO_2-x,_ NiO_x_, etc. [217, 218]. These molecules strongly chemisorb onto oxide surfaces through P–O–metal bonds, forming stable interface layers. The intrinsic dipole moment of the phosphonic acid group, combined with tunable functional end groups, enables precise control over surface work function and band alignment [219, 220]. In addition to dipole formation, these molecules effectively passivate surface defects such as oxygen vacancies and hydroxyl groups, further reducing interface recombination. Their strong binding and chemical stability make them suitable for tandem architecture, where long-term durability under illumination and thermal stress is essential. Thus, interface dipole engineering leads to several key improvements: reducing band offsets at the perovskite/transport layer interface, enhancing the built-in electric field to improve charge separation, and suppressing carrier accumulation at critical interfaces. Chen et al. developed an electric dipole-oriented defect healing strategy using sulfur-based 3-sulfopropyl acrylate/methacrylate potassium salts (SPA/SPM) as dual-channel molecular modulators for WBG PSCs [221]. The SPA and SPM molecules contain oxygen-rich C═O and −SO_3_^−^ functional groups that effectively coordinate with undercoordinated Pb^2+^ defects, thereby enabling simultaneous defect passivation and interface dipole alignment. As shown in Fig. 7a, SPM exhibited a larger molecular dipole moment than SPA owing to the additional −CH_3_ group, which altered the molecular self-assembly behavior at the perovskite interface. Density functional theory calculations further revealed that the oxygen atoms from the C═O and −SO_3_^−^ groups donate lone-pair electrons to Pb^2+^, forming stable Pb–O bonds and effectively suppressing defect states. The adsorption configurations illustrated in Fig. 7b and c demonstrated that perpendicular adsorption geometries were energetically more favorable than parallel configurations, particularly when the dual-site coordination originated from the −SO_3_^−^ group. Such perpendicular adsorption promoted the formation of oriented interface dipoles, facilitating enhanced charge separation and carrier transport. Furthermore, ToF–SIMS analysis (Fig. 7d) confirmed the accumulation of −SO_3_^−^ groups at both the top and buried interfaces of the perovskite films, verifying the formation of oriented dipole layers. As schematically illustrated in Fig. 7e and Fig. 7f, the aligned molecular dipoles generated a permanent interface electric field at the perovskite/C_60_ interface, which facilitated electron extraction, suppressed interface non-radiative recombination, and mitigated V_OC_ losses in WBG PSCs. Together, these effects translate into high V_OC_, improved FF, and reduced hysteresis. The integration of SAMs, fullerene derivatives, and phosphonic acid molecules provides a comprehensive toolkit for controlling interface energetics and suppressing recombination losses. As tandem solar cell architecture continues to evolve, the precise design and implementation of interface dipoles will play an increasingly important role in reducing the gap between theoretical limits and practical performance.

#### Low-temperature processing constraints for tandem integration

The fundamental requirement for integrating WBG perovskite top cells with CIGS bottom cells is low-temperature processing. The underlying CIGS absorbers and their buffer layer can undergo interdiffusion at elevated temperatures. Thus, the entire WBG perovskite top cell stack must be processed below ~150 °C to 200 °C, imposing a stringent thermal budget on all subsequent processing steps. This limitation significantly constrains crystallization kinetics, defect annealing, and film densification in both the perovskite absorber and the charge transport layers, often resulting in elevated defect densities. In particular, inorganic ETLs, such as SnO_2_ and TiO_2_, tend to exhibit higher oxygen vacancies, structural disorder, and hydroxyl groups, which contribute to interface traps at critical interfaces.

Plasma-based processes involving high-energy ions and UV radiation, which are widely used for surface activation, cleaning, or deposition (i.e., sputtering of TCO), induce damage to the perovskite surface by breaking chemical bonds and accelerating ion migration. Plasma-induced damage can create defects at the critical interface and increase the V_OC_ deficit [222, 223]. Further, solvent compatibility is another important constraint in the sequential deposition of the CIGS/perovskite tandem cells. Most of the organic solvents used to deposit charge transport layers or interface modifiers can dissolve or damage the absorber surface [224]. Additionally, solvent penetration can lead to ion redistribution and defect formation at the buried interface [225]. Sequential deposition also imposes limitations on material selection, deposition techniques, and process conditions. Sputtering of transparent conductive oxides or deposition of recombination layers can introduce mechanical stress, ion bombardment, or thermal loading, all of which can degrade the critical interfaces. Therefore, careful process engineering, such as the use of low-temperature ALD, soft deposition techniques, robust interface passivation layers, and solvent orthogonality, is essential to minimize defect formation and ensure stable, high-performance operation of CIGS/perovskite tandem solar cells. The long-term operational stability of WBG perovskite top cells is strongly influenced by the density and chemistry of interface defects. Electronic trap states located at the perovskite/charge-transport-layer interfaces promote trap-assisted SRH recombination and facilitate the accumulation of mobile ionic species under continuous illumination, thereby accelerating V_OC_ degradation and photo-induced halide segregation [203, 204]. First-principles calculations further demonstrate that surface and interface defects possess lower defect formation energies than the bulk, making these regions energetically favorable pathways for ion migration and defect accumulation during prolonged device operation [204]. Consequently, effective interface passivation suppresses defect-assisted ion migration, retards halide redistribution, and stabilizes the quasi-Fermi level splitting. Quantitative operational stability studies further demonstrate that interface engineering substantially prolongs device lifetime. For example, Yang et al. reported that interface passivation enabled n–i–p perovskite solar cells to retain over 90% of their initial efficiency after 700 h of continuous maximum-power-point operation, while simultaneously suppressing interface recombination and enhancing the FF [226]. More recently, Deng et al. demonstrated that dual-interface synergistic passivation achieved a T90 lifetime exceeding 700 h while maintaining a certified PCE above 25%, confirming that reducing interface defect density markedly improves operational durability [227]. Collectively, these studies establish interface defect density and chemistry as key quantitative descriptors governing V_OC_ degradation, defect-assisted ion migration, and the long-term operational stability of WBG perovskite solar cells.

## Tandem specific integration challenges

**Fig. 8:**
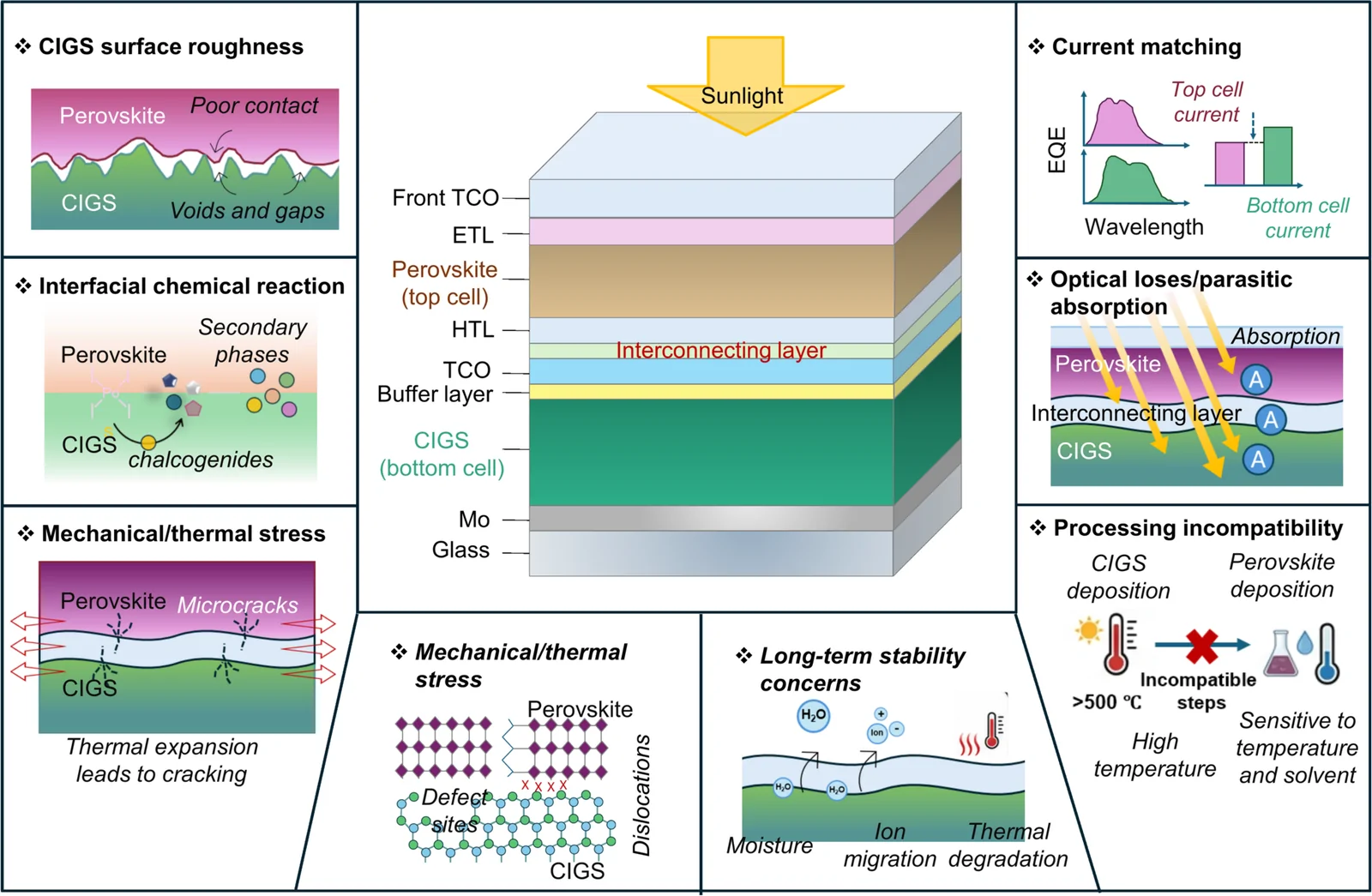
Schematic representation of the tandem-specific integration challenges for a 2 T CIGS/perovskite tandem solar cell highlighting surface and interface defects

The most important limitations are not associated with the absorber materials alone, but rather with the interfaces and connecting layers that govern charge transfer, voltage preservation, and long-term stability. Three challenge domains repeatedly emerge in the literature. First, interface defects arise from morphological mismatch, chemical incompatibility, and imperfect wetting when a solution-processed perovskite top cell is integrated on a rough CIGS bottom stack. Second, recombination dynamics at the interconnection layer determine whether the two subcells behave as an efficient monolithic series-connected device or suffer avoidable voltage and FF losses. Third, stability remains strongly limited by stress-assisted defect generation, ion migration, phase segregation, and environmental degradation pathways, many of which are initiated or accelerated at interfaces. Figure 8 shows the schematic representation of the tandem-specific integration challenges for a 2T CIGS/perovskite tandem solar cell. The discussion is organized into four major sections covering interface defect formation, recombination-layer design, stability implications, and future research opportunities.

### Interface defects in CIGS/perovskite tandem integration

#### Surface roughness and morphological incompatibility

The first major obstacle in monolithic CIGS/perovskite tandem integration is the morphological incompatibility between the rough CIGS bottom cell and the comparatively defect-sensitive perovskite top absorber. CIGS absorbers and their overlying contact stacks commonly present substantial microscale and nanoscale roughness. Reported surface root-mean-square roughness (σRMS) values for CIGS absorbers generally lie in the range of 50–200 nm, with typical lateral feature sizes from approximately 500 nm to 1 µm [7, 228, 229]. In the work of Jost et al., the relevant bottom CIGSe cell surface exhibited σRMS ≈ 75 nm, a magnitude already sufficient to complicate conformal deposition of subsequent layers [7]. Such topography differs fundamentally from smooth substrates typically used for high-performance perovskite processing and immediately introduces risks associated with thickness nonuniformity, incomplete coverage, and local electric-field enhancement [230]. This roughness problem is especially severe for solution-based perovskite deposition methods such as spin-coating. These approaches rely on controlled wetting, solvent evaporation, and nucleation over relatively uniform surfaces. When the underlying CIGS stack contains ridges, valleys, and abrupt local slopes, wet-film thickness becomes spatially heterogeneous and drying kinetics become position-dependent [231]. The consequence is that perovskite precursor films can dewet from elevated features or accumulate excessively in recessed regions, preventing formation of a laterally uniform crystalline top perovskite layer.

Beyond alkali engineering, partial substitution of Cu with Ag has emerged as an effective strategy for improving CIGS absorber quality. Ag incorporation promotes larger grain growth, enhances surface smoothness, and suppresses the formation of detrimental secondary phases, resulting in improved junction quality and reduced interface recombination [232, 233]. In addition to these structural benefits, Ag also influences Ga distribution and the resulting bandgap grading profile within the absorber. As discussed in Sect. 2.4, alkali elements play a critical role in defect passivation and interface engineering; notably, Ag incorporation can further modify the diffusion behavior and spatial distribution of alkali species such as Na and K. The interplay between Ag alloying, compositional grading, and alkali redistribution provides an additional degree of freedom for tailoring absorber microstructure, interface energetics, and defect chemistry, offering a promising pathway toward higher-performance CIGS and monolithic CIGS/perovskite tandem solar cells [234, 235].

Chemical incompatibility compounds the topographic challenge. ZnO layers commonly used in the bottom CIGS cell or recombination-contact region are chemically alkaline and can react unfavorably with the deposited perovskite when the interconnection layer lacks sufficient compactness. Imperfectly dense interfaces therefore create not only a geometric problem but also a chemical one, because local exposure of reactive ZnO can trigger decomposition or defect formation from the contact interface during top cell fabrication, especially under thermal stress [8, 236]. Over longer timescales, the same nonuniform interface environment promotes irregular perovskite nucleation and growth, since nucleation and growth conditions govern defect density and prevalence, and the large fraction of resulting GBs introduces surface and structural trap states [237]. The implication is that roughness is not merely a manufacturing inconvenience; it is an initiating factor for a cascade of interface defects that ultimately reduce V_OC_ through enhanced non-radiative SRH recombination, suppress FF, and accelerate long-term degradation, with trap passivation at GBs and interfaces remaining central to mitigating the V_OC_ deficit characteristic of wide-bandgap perovskite top cells integrated on rough bottom CIGS cells [238, 239].

#### Mechanical stress at the interconnection interface

Recent work has shown that the role of roughness extends beyond coating uniformity and directly influences the mechanical stress state of the integrated perovskite layer [27, 240]. In early reported tandem devices, the smoothness of the ICL was recognized as crucial for creating reliable contact between the two subcells, given that the planar perovskite top cell comprises several functional layers with thicknesses ranging from tens to hundreds of nanometers that are sensitive to substrate roughness. Strategies such as chemical mechanical polishing of the Indium tin oxide (ITO) recombination layer were adopted to address this challenge [8], though such approaches proved costly and difficult to scale for commercial production [241]. More recently, the development of the CIGS/perovskite tandem technology has been persistently slowed by the rough surface of the bottom CIGS subcell, which induces macroscopic defects in the cell stack originating from the fabrication process [12].

Pei et al. demonstrated that CIGS substrate roughness modulates perovskite film stress in a quantitatively significant manner. Smooth CIGS substrates induce tensile stress in the overlying perovskite film, which weakens bonding interactions, induces chemical structural instability, and lowers both defect formation energy and ion migration activation energy, impeding long-term stability under photothermal stress. Rough CIGS substrates featuring corrugated surface morphology effectively alleviate these harmful tensile stresses, enhancing photothermal tolerance and optoelectronic properties in the corresponding perovskite films. Specifically, smooth substrates with Rq = 31.3 nm induced tensile stress of 17.52 MPa, whereas rougher corrugated substrates with Rq = 90.3 nm reduced this to 2.75 MPa, an 84.3% reduction [240]. This result is initially counterintuitive, as rougher substrates are often assumed to be mechanically less favorable; however, in this system the corrugated morphology evidently allows stress relaxation more effectively than a flatter but mechanically constraining interface [240].

The relationship between residual tensile stress and perovskite instability is not unique to the CIGS/perovskite system but reflects a broader and well-established phenomenon. Thermally induced tensile strain that remains in perovskite films following annealing is a known source of instability, as it weakens bonds, decreases the formation energy of defects, and lowers the activation energy for ion migration. Strain leads to lattice distortion that alters the electronic band structure, and the resulting deeper defect levels can act as non-radiative recombination centers that degrade device performance [242]. This is especially problematic for WBG mixed-halide perovskites, where strain inhomogeneity accelerates ion migration and increases susceptibility to phase segregation [108, 243]. Consequently, when defect formation energy is lowered by tensile stress at the CIGS/perovskite interface, vacancies, interstitials, and other electronically detrimental defects emerge more readily under thermal or photothermal operation [244]. Simultaneously, reduced ion migration activation energy promotes halide redistribution and compositional instability [240].

In the tandem context, stress-modulated defect formation has immediate consequences for both device efficiency and reliability. A mechanically stressed interface is more likely to contain electronically active states that enhance SRH recombination, disturb band alignment, and create local current bottlenecks [242]. Moderate stress can cause dislocations or defects at the interface that enhance recombination of photogenerated carriers, and stress can simultaneously facilitate light-induced phase segregation of wide-bandgap perovskite, leading to bandgap changes and further performance loss [245]. Over time, the same stressed zones can evolve into nuclei for crack formation and delamination. Thermal stress from mismatched thermal expansion coefficients can reach as high as 50 MPa in perovskite films and become a driving force for film deformation, ion migration, and eventual delamination of the layered structure [246]. Cross-sectional analysis of thermally cycled devices has revealed two types of irreversible morphological degradation features at the interface: cracks within the perovskite grains and delamination at the perovskite/charge transport layer boundary [247]. Therefore, the interface stress problem should be understood as a bridge between initial integration quality and later operational degradation, linking morphology, lattice stability, and defect kinetics within a single failure framework [240].

#### Interface defect passivation strategies

**Fig. 9:**
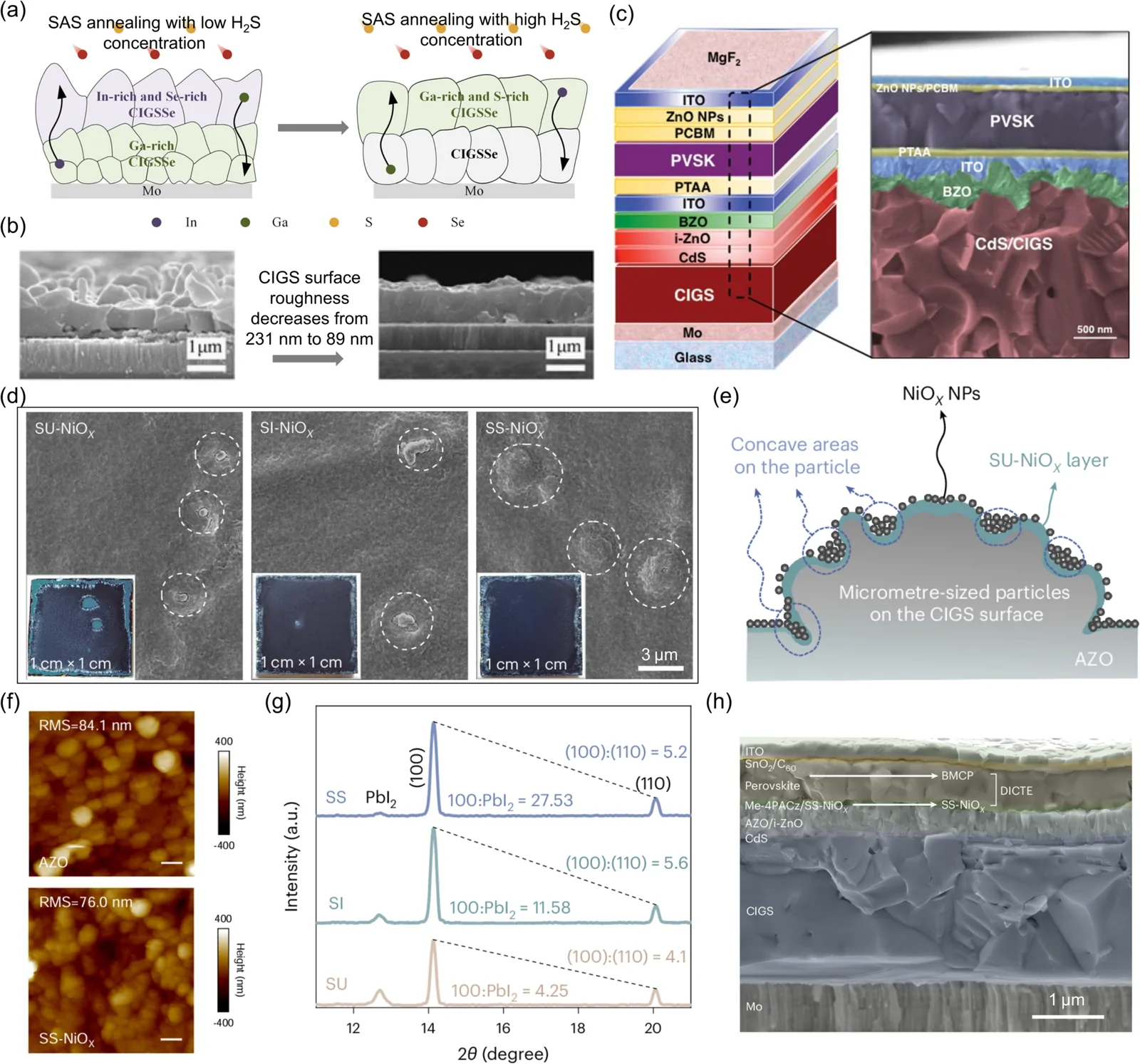
Reported strategies to mitigate surface and interface defects in 2 T CIGS/perovskite tandem solar cells. **a** Schematic representation of the SAS annealing process for the CIGSSe absorber to reduce surface roughness, **b** corresponding cross-sectional SEM images of the CIGSSe absorber before and after SAS annealing. Reproduced with permission from Ref. [248] **c** 2 T device structure with cross-sectional SEM image of the chemical mechanical polishing process of ICL. Reproduced with permission from Ref. [8]. **d** Surface SEM images of perovskite films on CIGS bottom cells with SU-NiO_x_, SI-NiO_x_, and SS-NiO_x_. Insets show photographs of the corresponding CIGS/AZO substrates. **e** Schematic diagram of SS-NiO_x_ deposited on a micrometre-sized particle. **f** Atomic force microscope (AFM) images and σRMS roughness of reference Mo/CIGS/CdS/i-ZnO/AZO and optimized Mo/CIGS/CdS/i-ZnO/AZO/SS-NiO_x_ different films, **g** Enlarged XRD spectra of perovskite films on SU-NiO_x_, SI-NiO_x_ and SS-NiO_x_, with corresponding intensity ratios of (100)/PbI_2_ and (100)/(110), and **h** Structure diagram of 2 T CIGS/perovskite TSCs with the DICTE strategy. Reproduced with permission from Ref. [13]

Because interface defects dominate losses in many present tandem devices, hole-transport layer engineering and self-assembled monolayer design have become central strategies for passivation. Recently, Hao et al. reported a sulfurization after selenization (SAS) annealing process for CIGSSe absorber to reduce surface roughness, as shown in Fig. 9a, b. By optimizing H_2_Se and H_2_S concentrations, the diffusion and distribution of In, Ga, S, and Se were controlled to achieve excellent crystallinity, low surface roughness, and a U-shaped bandgap. The optimized process reduced surface roughness by 58.2%, with Rq values decreasing from 231 to 89 nm [248]. Mechanical polishing of ICL is another great strategy reported to reduce the bottom cell roughness, as shown in Fig. 9c**.** One of the key demonstrations was reported by Jošt et al., who used conformal ALD NiO_x_ on rough CIGS surfaces with σRMS around 75 nm. The conformal nature of ALD NiO_x_ suppressed microscale shunt paths that otherwise arise when the rough substrate is covered only by less conformal organic transport layers. The effect of this approach is evident from the strong dependence of tandem performance on the interface stack composition. In this work, PTAA alone yielded poor device metrics, with V_OC_ around 0.56 V, FF near 35%, and efficiency of approximately 3.1%. By contrast, the NiO_x_/PTAA combination enabled a PCE of 21.6%, V_OC_ of 1.58–1.59 V, and FF of 75.7–76.0% [7]. The role of NiO_x_ is not limited to energy-level positioning. Its conformality reduces local electrical shorts, improves perovskite nucleation on rough surfaces, and provides a more chemically inert and mechanically robust interface.

A representative solution to the roughness problem outlined above could be the dual-interface carrier transport engineering (DICTE) strategy consisting of a nanoparticle-assisted bilayer NiO_x_ hole-transport architecture recently reported by Zeng et al. [13]. Rather than relying on a single sputtered or solution-processed NiO_x_ layer, the authors sequentially deposit a sputtered NiO_x_ film (SU-NiO_x_) followed by spin-coating a thin layer of NiO_x_ nanoparticles (SI-NiO_x_), then crystallize the stack by air annealing to form what they term SS-NiO_x_. Plan-view SEM confirms that the nanoparticle layer selectively fills the concave regions between micrometre-scale asperities on the textured CIGS/AZO surface that the sputtered layer alone cannot access, as shown in Fig. 9d. SU-NiO_x_ leaves exposed AZO tips and permits pinhole formation in the perovskite overlayer, whereas SS-NiO_x_ achieves conformal, largely void-free coverage (Fig. 9e). This translates into a measurable reduction in substrate roughness (σRMS from 84.1 to 76.0 nm) (Fig. 9f) and, downstream, a smoother, more compact perovskite film with a stronger (100) crystallographic orientation (XRD (100):(110) ratio increasing by a factor of 4.1–5.6 relative to SU-NiO_x_) and substantially suppressed residual PbI_2_ (the (100)/PbI_2_ ratio rising from 4.25 to 27.53), as shown in Fig. 9g. The reduction in reverse dark leakage current by more than three orders of magnitude (1.61 × 10^–3^ to 8.42 × 10^–7^ mA cm^−2^) reported by Zeng et al. provides direct electrical evidence that improved conformality translates into suppressed shunting pathways, reinforcing the point that roughness-induced defect formation in CIGS/perovskite tandems is not solely a geometric problem but one with direct consequences for non-radiative recombination and device yield [13]. Finally, Champion monolithic tandem cells (Fig. 9h) achieve PCE of 31.09% (certified 30.57%, steady-state 30.32%) for small-area devices (0.0539 cm^2^) and 29.44% (certified 28.85%) for larger-area devices (1.0298 cm^2^) [13].

Taken together, these studies show that defect passivation in CIGS/perovskite tandems requires multiscale control. A successful strategy must simultaneously address geometric conformity on rough CIGS, chemical compatibility with oxide contacts, reduction of interface trap density, and favorable energy-level alignment. No single layer solves all of these issues independently. Instead, high-performing interfaces are achieved by combining inorganic conformal coatings, organic hole-transport modifiers, and molecular monolayers selected for both passivation strength and electronic matching [7, 14, 235].

### Recombination of junction and interconnecting layer defects

#### Architecture and function of interconnection layers

**Fig. 10:**
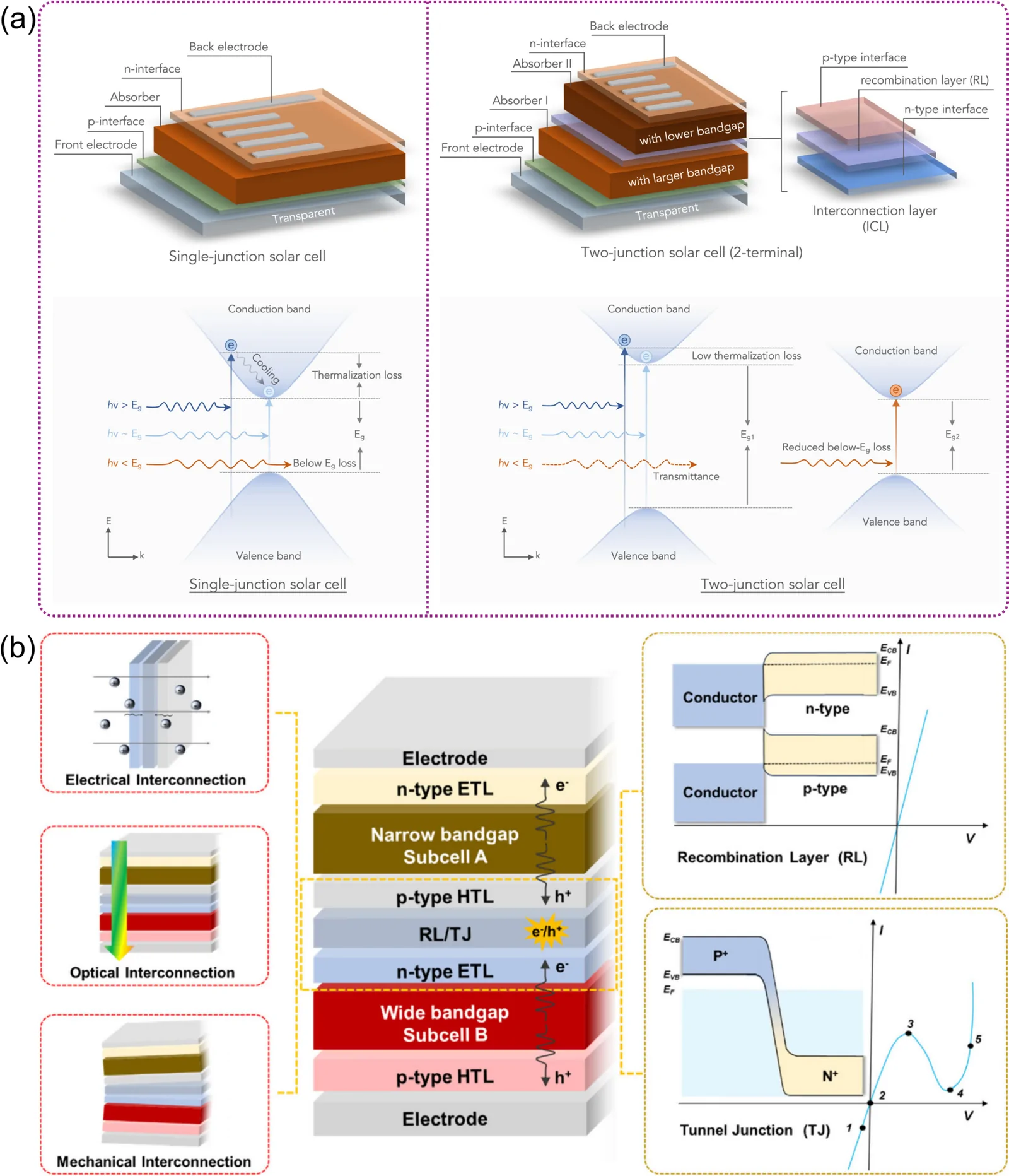
**a** Device architectures of single-junction and two-junction (tandem) solar cells and band diagram showing the two main intrinsic loss mechanisms: thermalization loss, where photons with hν > *E*_g_ generate hot carriers that lose excess energy as heat upon relaxation to the band edge, and sub-bandgap loss, where photons with hν < *E*_g_ are transmitted without absorption. Reproduced with permission from Ref. [249]. **b** Role of the ICL in tandem solar cells: the ICL facilitates charge extraction from adjacent subcells while providing recombination sites for electron–hole pairs at the internal junction. Tailoring the work function difference between the n-type and p-type layers of the ICL mitigates the Schottky barrier and promotes faster charge transfer. Reproduced with permission from Ref. [251]

The comparison between single-junction and 2T tandem solar cells is shown in Fig. 10a. In 2T monolithic tandems, the ICL serves as the electrical and optical bridge between the subcells and is therefore one of the most consequential elements in the stack [249]. The recombination layer, or intermediate recombination layer (IRL), connects the two photovoltaic junctions in series, so the total tandem voltage is ideally the sum of the subcell voltages, whereas the externally flowing current equals that of the subcell with the lowest generated current [249, 250]. Table 3 shows the reported state-of-the-art 2 T CIGS/perovskite tandem solar cell with different IRL layers and defect passivation strategies. As shown in Fig. 10b, the ICL must simultaneously provide efficient electrical coupling, sufficient optical transparency, and robust suppression of parasitic leakage pathways [251]. The physical implementation of the ICL differs substantially across material platforms: epitaxial III–V multijunctions employ degenerately doped p++/n++ tunnel junctions that rely on direct band-to-band tunneling; thin-film Si tandems employ defect-mediated recombination at doped amorphous or nanocrystalline Si interfaces; and perovskite-based tandems, whether paired with Si or with a second perovskite absorber, employ sputtered or ALD-deposited transparent conductive oxides such as ITO or IZO, occasionally supplemented with ultrathin metal or metal-nanoparticle interlayers [252]. From an electronic standpoint, the desired function is efficient majority-carrier recombination at the junction, often described in terms of band-to-band tunneling or direct tunneling through a sufficiently thin and well-aligned interlayer. At the same time, minority carriers should be blocked to prevent leakage currents and maintain high FF [249]. This dual requirement is difficult because materials that facilitate conductivity and recombination can also introduce absorption, roughness sensitivity, or poor selectivity. As a result, the ICL is rarely ideal in current CIGS/perovskite tandems and frequently acts as a hidden source of V_OC_ and FF loss [238]. Conversely, an optimally designed ICL should exhibit conductivity σ > 10^3^ S/cm to minimize series resistance, optical transmittance > 90% in the perovskite absorption range (400–800 nm), and work function alignment that generates near-equilibrium recombination across a minimal distance (~ nm scale) to reduce minority-carrier diffusion losses [253, 254].

**Table 3:** Comparison table of reported state-of-the-art 2T CIGS/perovskite tandem solar cells with different IRL layers and defect passivation strategies

Author (year)	Perovskite Composition (*E*_g_)	CIGS *E*_g_	IRL	Defect Passivation Strategy	V_OC_ (V)	FF (%)	PCE (%) [Ref.]
Farias-Basulto et al. (2025)	Cs_0.05_(MA_0.17_FA_0.83_)_0.95_Pb(I_0.83_Br_0.17_)_3_(1.63 eV)	1.08 eV	NiO_x_:Cu + SAM (MeO-2PACz) HTL /C_60_/SnO_x_ ETL	NiO_x_:Cu–SAM bilayer for low quasi-Fermi-level splitting–V_OC_ offset	1.675	72.7	23.14 [255]
Geng et al. (2025)	Cs_0.05_FA_0.8_MA_0.15_Pb(I_0.8_Br_0.2_)_3_ (Not stated)	Not stated	R-SAM (I-2PACz-reconstructed Me−4PACz) + BSSS (F5BPA/p-CF3PEAI) at PVK/C_60_	Dual interface engineering: SAM reconstruction (NiO_x_/PVK) + bimolecular surface self-accumulation (PVK/C_60_)	1.726	75.4	25.21 [256]
Farias-Basulto et al. (2025)	Cs_0.05_(MA_0.1_7FA_0.83_)_0.95_Pb(I_0.83_Br_0.17_)_3_ (1.63 eV)	1.05–1.06 eV	NiO_x_ nanoparticle + 2PACz SAM HTL	Bandgap grading (lower deposition T) + thinner IZO for optical/current matching	1.765	71.8	24.6 [257]
Pei et al. (2025)	DMA_0.1_Cs_0.25_MA_0.05_FA_0.6_Pb(I_0.9_Br_0.1_)_3_ (1.67 eV)	Not stated	NiO_x_/SAM HTL; interconnection stress relief via corrugated (rough) CIGS	Rough-CIGS interconnection contact relieves perovskite tensile stress; bulk/surface bimolecular passivation	1.946	76.47	28.02 [240]
Ying et al. (2025)	Cs_0.17_FA_0.83_(PbI_0.4_Br_0.6_)_3_ (1.65 eV)	1.1 eV	Me−4PACz SAM (antisolvent-seeding strategy)	Decoupled SAM dissolution/adsorption + pre-mixed perovskite seed layer for high-density, uniform SAM	1.75	77.2	23.8 [258]
Zhou et al. (2025)	Cs_0.05_MA_0.15_FA_0.8_Pb(I_0.75_Br_0.25_)_3_ (1.67 eV)	Not stated	NiO_x_/Me-SAF (spiro-type SAM) HTL bilayer	3D π-conjugated Me-SAF suppresses π–π stacking/molecular aggregation vs. planar Me−4PACz	1.85	76.7	25.5 [259]
Li et al. (2026)	(FA_0.8_Cs_0.2_)Pb(I_0.82_Br_0.15_Cl_0.03_)_3_ (1.67 eV)	1.01 eV	AZO/Au/NiO_x_/4PADCB composite IRL	4PADCB SAM passivates NiO_x_ surface traps; ultrathin Au boosts majority-carrier recombination	1.731	80.2	30.1 [14]
Zeng et al. (2026)	Cs_0.05_(FA_0.83_MA_0.17_)_0.95_Pb(I_0.8_Br_0.2_)_3_ (1.65 eV)	1.01 eV	AZO/SS-NiO_x_/Me−4PACz composite IRL	Dual-interface carrier transport engineering addressing CIGS/ICL and perovskite/C_60_ interfaces	1.765	83.11	30.57 [13]

#### Defect formation at the ICL

Defect formation at the ICL arises through mechanisms that are largely absent from single-junction device fabrication, because the ICL is deposited directly onto, and interfaces with layers belonging to two independently optimized subcells. However, TCO-based intermediate layers have several limitations, including sputtering-induced damage to the underlying cell, current shunting due to their high lateral conductivity, and free-carrier parasitic absorption that reduces the photocurrent of the bottom subcell [252]. A further source of defect formation is the incompatibility between the thermal budgets of the two subcells, which frequently constrains ICL deposition to low-temperature conditions and results in incomplete crystallization, pinhole formation, and elevated GB trap densities. Mobile ionic species, including halide ions and organic cations in perovskite absorbers, migrate toward the internal interface under combined bias and illumination, generating deep trap states and, in some cases, reacting with the TCO to form additional non-radiative recombination centers [260]. In epitaxial III–V systems, the heavy doping required to sustain tunneling promotes dopant diffusion and the formation of misfit dislocations at the tunnel-junction interface, both of which introduce parasitic recombination pathways that compete with the intended tunneling process. Additionally, in perovskite/Si tandems, the pyramidal surface texture employed for light trapping in the silicon bottom cell complicates conformal deposition of the ICL and overlying top cell layers, resulting in localized shunting and regionally elevated defect densities at pyramid apexes and valleys [260, 261].

#### Recombination losses related to the ICL

Recombination losses associated with the ICL likewise diverge from those characteristic of single-junction absorbers. In epitaxial tunnel junctions, band-to-band tunneling dominates, with its efficiency governed exponentially by depletion width and thus requiring doping densities more than approximately 10^19^ to 10^20^ cm^−3^; the peak sustainable current density of such junctions is further constrained by Auger-assisted tunneling processes. In amorphous silicon and TCO-mediated junctions, trap-assisted tunneling through mid-gap defect states provides the dominant recombination pathway. Notably, unlike single-junction devices, in which defect-mediated recombination is uniformly parasitic, the ICL frequently requires a controlled density of recombination centers to function at all, since an insufficient density results in blocking behavior and current rollover, while an excessive density increases parasitic absorption and resistive loss. Parasitic optical absorption within the ICL itself, arising from free-carrier absorption in heavily doped tunnel-junction layers or in the TCO, constitutes an additional loss channel with no equivalent in single-junction architectures, as does the resistive voltage drop introduced across the junction, which adds directly to overall device loss in FF and V_OC_ [262].

Collectively, these considerations distinguish the monolithic 2T tandem from the single-junction device in several respects: the presence of two absorber junctions bridged by an internal recombination junction rather than a single junction connected directly to external contacts; a series current-matching constraint absent from single-junction operation; the placement of an internal, optically active layer within the light path that introduces parasitic absorption; the requirement to reconcile two independently optimized thermal and chemical processing windows at a single shared interface; and failure signatures, including tunneling breakdown and current rollover, that reflect the additional electrical and optical complexity introduced by the recombination junction itself.

#### Traditional and alternative recombination materials

The ITO remains the most widely used recombination-junction material in tandem photovoltaics because of its high conductivity and transparency. However, its high lateral conductivity can become a liability in defective devices. When top cell regions contain local shunts or poorly passivated defects, lateral current pathways through ITO can bypass the intended local series-connection behavior. This amplifies the electrical influence of small defective zones and can reduce effective V_OC_ and FF at the device level. In rough or nonuniform tandem stacks, this problem is particularly relevant because shunt-prone areas are more likely to occur. As a replacement for ITO, indium zinc oxide (IZO) is considered a suitable ICL at the CIGS/perovskite interface due to its superior NIR transmittance (~ 80% above 800 nm) compared to ITO (~ 70%), allowing more photons to reach the CIGS bottom cell and increasing J_SC_ by 1–3 mA cm^−2^ [251].

**Fig. 11:**
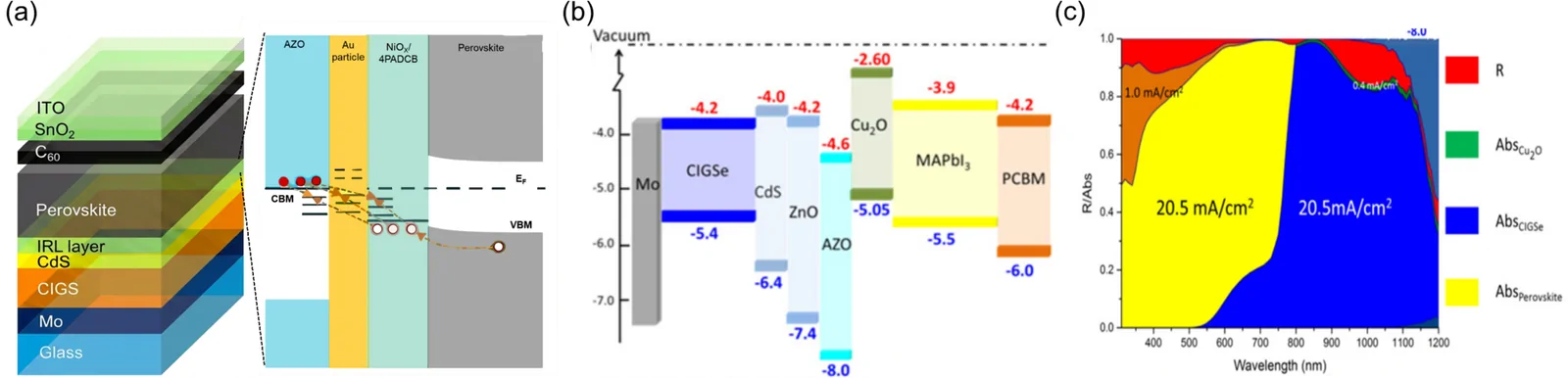
**a** Schematic representation of modified IRL and the energy level diagram of the AZO/Au/NiO_x_/4PADCB recombination junction. Reproduced with permission from Ref. [14] **b** Energy-level and structure diagrams of a CIGS/perovskite tandem solar cell relative to the vacuum level, values in red are the conduction band minimum, while values in blue are the valence band maximum, **c** reflection and absorption of the whole tandem device under the condition of matched current density between top perovskite and bottom CIGS cell. Reproduced with permission from Ref. [263]

Alternative and composite interlayers have also attracted increasing attention. Li et al. reported a composite IRL design based on AZO/Au/NiO_x_/4PADCB, in which insertion of an ultrathin 0.6 nm Au layer at the AZO/NiO_x_ interface significantly improved junction behavior (Fig. 11a). In addition, the composite IRL suppresses photo-induced segregation in the perovskite layer and enhances the stability of unencapsulated tandem devices. The device fabricated by Li et al. has demonstrated increased FF from 71 to 76%, V_OC_ from 1.71 V to 1.73 V, and PCE from 25.6% to 27.6% [14]. The proposed mechanism was that Au promotes majority-carrier, specifically hole, recombination while reducing minority-carrier electron accumulation at the junction [14]. Such a design illustrates how an intermediate layer can be engineered not merely for conductivity but for selective recombination kinetics. Han et al. reported a high-efficiency CIGS/perovskite with V_OC_ of 1.774 V and the PCE of 22.43% by employing the ICLs of CdS/i-ZnO/BZO/ITO/PTAA [8].

#### Band alignment and energy-level matching

Band alignment and energy-level mismatches at the multiple interfaces of 2 T CIGS/perovskite tandem devices are a primary source of defect-mediated recombination and V_OC_ losses. The energy-level and structure diagrams of a perovskite-CIGS tandem solar cell relative to the vacuum level are shown in Fig. 11b. Energy-level alignment requirements at the ICL differ fundamentally from those governing single-junction absorber–contact interfaces, in that the ICL must satisfy alignment conditions for two carrier types originating from two distinct subcells rather than a single alignment condition for one carrier type extracted to one external contact. In directly recombining junctions, the conduction band, or electron quasi-Fermi level, of the n-type layer feeding the junction must lie near the valence band, or hole quasi-Fermi level, of the adjacent p-type layer to permit recombination with minimal energetic penalty [251, 264]. In TCO-mediated junctions, where the TCO behaves in a quasi-metallic manner owing to its high free-carrier density, the critical alignment condition shifts to the two separate ohmic contacts formed between the TCO and each adjacent transport layer, both of which must be engineered, often through the insertion of thin interlayers, to minimize extraction barriers. Energetic misalignment at either interface manifests as an S-shaped kink or current rollover in the illuminated current–voltage characteristic, a signature of internal junction impedance that is not observed in the well-behaved diode characteristics of single-junction devices [251]. The IRL (ITO or AZO) in combination with HTLs like NiO_x_ or SAMs must simultaneously satisfy charge-selective transport, work function matching, and minimal trap-state density. Any mismatch in energy level alignment, charge extraction inefficiencies, or interface defects can severely impact V_OC_ and overall PCE, making interface design even more critical in tandem architectures than in single-junction devices, due to the presence of multiple stacked interfaces [238]. Han et al. reported that, in CIGS/perovskite tandem device, the BZO or ZnO front contact of the CIGS subcell presents a significant work function mismatch: the BZO work function of approximately −4.0 eV is substantially lower than that of PTAA (~ −5.1 eV), creating a large contact potential barrier at this interface, requiring an ITO interlayer to modify the surface work function and establish a better ohmic contact for hole transport [8].

At the NiOₓ/perovskite interface, defect passivation is equally critical because NiOₓ surface defects and misaligned energy levels reduce interface hole extraction and require treatment with dipole-bearing SAM molecules such as 4PADCB to passivate trap states, reduce interface recombination, and restore favorable energy level alignment [14]. The dominant loss mechanism tied to these defects is SRH recombination directly inducing V_OC_ losses that limit device efficiency [238]. Therefore, in monolithic CIGS/perovskite tandems, mismatched band gaps and the lack of efficient IRLs are identified as the primary bottlenecks, reflecting the tremendous challenges that remain despite years of development.

#### Current matching and spectral response

Current matching is a fundamental constraint in monolithic 2 T CIGS/perovskite tandem devices, where the series-connected subcells are limited by whichever delivers the lower J_SC_. Because subcells are series-connected, the J_SC_ of the tandem is limited by whichever subcell generates the lower current; any imbalance forces the current-limiting subcell to operate below its optimal point, directly reducing overall power output and making current matching a primary design constraint [239], as shown in Fig. 11c. Achieving current matching requires coordinated optimization of perovskite bandgap, absorber thickness, TCO optical properties, and CIGS composition. At higher perovskite bandgaps, insufficient light absorption in the top cell prevents current matching with the CIGS subcell, while lower bandgaps sacrifice V_OC_; EQE measurements of the certified 24.2% device revealed a residual 1 mA cm⁻^2^ current density mismatch between subcells, identifying further optical improvement as a key path to higher efficiency [265]. The most effective strategy for resolving this imbalance has been to simultaneously lower the CIGS bottom cell bandgap and improve the optical stack of the perovskite top cell: in the certified PCE of 24.6% CIGS/perovskite tandem, the improvement in J_SC_ over the prior record was attributed primarily to lowering the bandgap of the CIGS bottom subcell from ~1.08–1.1 eV to 1.05–1.06 eV and improving the optics of the top perovskite cell [257]. TCO parasitic absorption in the NIR represents a persistent source of current loss to the CIGS subcell. ITO, while commonly used in perovskite tandems, exhibits significant free-carrier absorption in the NIR; higher-mobility alternatives such as IZO and hydrogen-doped indium oxide (IO:H) require lower doping concentrations to achieve equivalent sheet resistance and therefore display substantially lower parasitic NIR absorption [266]. This was demonstrated directly in the CIGS/perovskite context by Schultes et al., where IO:H and IZO electrodes reduced the total NIR absorptance (800–1300 nm) of the perovskite top cell to below 7%, maximising the photocurrent available to the CIGS bottom cell [266]. For the 2 T configuration, optical simulation combined with EQE measurements is the standard diagnostic tool: optical simulations using simulated annealing were applied to the certified 24.2% device to determine the optimal device stack, projecting a J_SC_ of 19.9 mA cm^−2^ and 32% PCE potential based on semiempirical material properties [265].

### Stability and reliability implications of defects

#### Long-term operational stability: T80 lifetimes

The T80 lifetime, defined as the time required for a device to degrade to 80% of its initial efficiency, is a standard metric for commercial viability. For crystalline silicon modules, T80 is ~20- 25 years under standard outdoor conditions [267, 268]. For CIGS/perovskite tandems to achieve commercial deployment in utility-scale and building-integrated applications, comparable T80 lifetimes (preferably > 10–15 years) are essential. Current research on CIGS/perovskite tandems reveals substantially shorter T80 lifetimes, typically below 1500 h under accelerated stress conditions (85 °C, 1000 W/m^2^ illumination) [240]. Extrapolating via Arrhenius models, assuming an activation energy of ~ 0.55–0.65 eV (typical for perovskite degradation), translates to T80 ~ 2–5 years under typical field conditions (average cell temperature ~ 45–50 °C). This gap between laboratory and field requirements necessitates substantial advances in material stability and device architecture [269].

Failure mode analysis via photoluminescence (PL) imaging, EQE measurements, and cross-sectional TEM has revealed multiple dominant degradation pathways: (1) perovskite decomposition to PbI_2_ and methylammonium lead iodide intermediates, manifesting as a broadened EQE response and reduced V_OC_; (2) interface defect growth, indicated by increasing dark saturation current and reduced FF; (3) TCO oxidation or reduction, degrading conductivity and increasing series resistance; and (4) CIGS layer chemical modification, including formation of oxides or secondary phases [270]. Recent stabilization efforts employing mixed-cation perovskites (Cs/FA/MA), 2D/3D heterophase structures, and advanced encapsulation have pushed T80 lifetimes toward 3000–5000 h under accelerated conditions (85 °C/85% RH), suggesting that extrapolated field T80 values approaching 5–8 years may be achievable [271]. However, further research, particularly on long-term field testing and on mechanisms of encapsulation failure (delamination, edge moisture ingress), is required to establish true commercial-grade durability.

#### Alternative stability pathways: extended thermal storage

A complementary picture of stability emerges from dark-storage and thermal-storage experiments on advanced interface chemistry. Li et al. reported that unencapsulated NiO_x_/4PADCB-based devices stored in an inert atmosphere at 25 °C retained 98.7% of their initial efficiency after 6600 h. At elevated-temperature storage at 65 °C for 678 h, efficiency retention remained about 86% [14]. These results indicate that sputtered NiO_x_ with molecular SAM capping can provide exceptional dark stability, likely due to stronger molecular anchoring to the oxide surface, denser monolayer packing, reduced moisture and oxygen penetration, and more stable perovskite/HTL interface chemistry. This stability pathway is distinct from the stress-relief mechanism highlighted by Pei et al. [240], but the two are conceptually compatible. One approach reduces the thermomechanical driving force for defect generation, while the other reduces the chemical and electronic susceptibility of the interface once formed. The broader implication is that long-term tandem reliability will probably require both strategies together: mechanically tolerant integration and chemically robust contact passivation.

#### Field performance and outdoor stability

While accelerated laboratory testing (thermal cycling, damp heat, thermal storage) provides standardized reproducible conditions for rapid failure-mode identification, outdoor field testing remains essential for validating true commercial readiness [272, 273]. Field deployment introduces stochastic variations in irradiance (cloud transients), temperature (daily and seasonal fluctuations), humidity (seasonal and microclimate-dependent variations), and mechanical stress (wind loading, thermal shock from sudden temperature drops during rain), which collectively drive degradation pathways not fully represented in laboratory tests [272, 274]. Field testing of CIGS/perovskite tandems remains sparse relative to other photovoltaic technologies, with most reports limited to pilot installations in controlled environments or short-duration (< 1 year) monitoring [255].

Farias‐Basulto et al. reported outdoor performance monitoring of perovskite-CIGSe tandem solar cells over one year of continuous operation in Berlin/Germany and revealed important insights into their practical viability and degradation mechanisms [255]. Starting with an initial efficiency of 23.14% before encapsulation, the tandem device showed reversible metastable behavior during outdoor operation, with performance initially improving during spring months before declining due to device degradation. At maximum outdoor performance, the tandem achieved instantaneous power output up to 68% higher than its single-junction CIGSe reference cell, with the peak performance occurring in April and thereafter decreasing due to degradation. The study employed multiple linear regression modeling to predict outdoor performance with accuracies exceeding 90%, and an online machine learning approach successfully tracked performance variations over time, reducing prediction errors despite metastability effects and environmental fluctuations. Importantly, layer delamination was identified as a major contributing degradation mechanism, highlighting the need for improved encapsulation and interface engineering to enhance the long-term commercial viability of perovskite-based tandem devices [255]. Table 4 shows the 2 T CIGS/perovskite tandem solar cells with reduced surface and interface defects and reported stability.

**Table 4:** Reported 2 T CIGS/perovskite tandem solar cells with reduced surface and interface defects

Year	2 T tandem device structure	PCE (%)	Note	Device stability	Ref
2015	Glass/Si_3_N_4_/Mo/CIGS/CdS/ITO/PEDOT:PSS/perovskite/PCBM/Al	10.9	Elimination of i-ZnO, as it degrades the perovskite layer	–	[275]
2017	Glass/Mo/CI(G)S/CdS/ZnO/ITO/PEDOT:PSS or Cu:NiO_x_:perovskite/C_60_/bis-C_60_	18.5	Improved spectral utilization	–	[276]
2018	Glass/Mo/CIGS/CdS/ZnO/ITO/ Cu:NiO_x_:perovskite/C_60_/bis-C_60_	22.4	Mechanical polishing of ITO; Novel i-ZnO/BZO/ITO ICL	88% after 500 h at 1 sun, 30 ℃ and 30% humidity	[8]
2022	Glass/Mo/CIGS/CdS/ZnO/SAM/perovskite/C_60_/SnO_2_/IZO/LiF	24.2	Refined interface engineering and current matching	Temperature analysis; Field performance	[265]
2022	Glass/Mo/CISe_2_/CdS/i-ZnO/IZO/NiO_x_/2PACz/perovskite/C_60_/ SnO_x_/IZO/Ag/MgF_2_	24.9	Bottom cell roughness and current matching	–	[106]
2025	Glass/Mo/CIGS/CdS/i-ZnO/ITO/NiO_x_/mixed-SAM/perovskite/C_60_/ SnO_x_/ITO/Ag/MgF_2_	28.65	Residual tensile stress improvement and suppression of nonradiative recombination: photothermal degradation dynamics	T80 lifetime (1123 h vs 400 h) under MPP tracking	[240]
2025	Glass/Mo/CISe_2_/InS/i-ZnO/IZO/NiO_x_/R-SAM/perovskite/C_60_/SnO_x_/IZO/Ag/LiF	26.14	Reconstruction of SAMs to tailor ETL interface properties	90.5% retention of initial stability after 50 days of air storage	[256]
2026	Glass/Mo/CIGS/CdS/i-ZnO/IZO/SS-NiO_x_/Me–4PACz/perovskite/C_60_/SnO_x_/ITO/Ag/ MgF_2_	30.57	Nanoparticle-assisted bilayer NiOₓ recombination layer + bimolecular co-passivation at perovskite/C₆₀	91.49% retention of initial stability after 756 h under MPPT tracking	[13]

### Challenges, opportunities, and future outlook

#### Current efficiency barriers and loss mechanisms

The best reported 2 T CIGS/perovskite tandem efficiencies demonstrate that the architecture is highly competitive but still significantly below its theoretical ceiling. Li et al. reported the highest in-lab efficiency of 30.71%, with the highest PCE of 30.10% (cross-verified by an external organization) [14]. Recently, Zeng et al. reported the certified PCEs of 30.57% and 28.85% for small-area (0.0539 cm^2^) and large-area (1.0298 cm^2^) 2 T CIGS/perovskite devices, respectively [13]. Meanwhile, the highest PCE for CIGS/perovskite tandem solar cells listed in the NREL Chart is 26.7% by the Korea Institute of Energy Research. This level represents substantial progress, yet it remains well below the approximate 45% tandem limit generally cited from S–Q considerations [277, 278]. The remaining performance gap is attributable to several coupled loss mechanisms. Interface nonradiative recombination is among the most important, often producing V_OC_ losses of about 100–150 mV per problematic subcell interface [14]. Optical losses arise from parasitic absorption in transparent conductive oxides and interlayers and from reflection or scattering at rough interfaces. Electrical losses originate from series resistance, contact resistance, and incomplete current matching [279–281].

Optical losses constitute a major barrier: the J_SC_ remains below 90% of the theoretical limit, with key losses arising from front reflection, Fresnel reflection, and parasitic absorption in interlayers. Parasitic absorption in TCO alone contributes 0.74 mA cm^−2^ of current loss, and reflective losses account for an additional 0.6 mA cm^−2^. Additionally, parasitic absorption losses in TCOs and broadband reflection losses at air/substrate interfaces account for a total current loss of 4–5 mA cm^−2^ [12, 257, 279, 282]. Nonradiative recombination losses, dominated by SRH recombination mechanisms, are equally critical: trap-assisted SRH recombination at interfaces is the dominant nonradiative process, inducing substantial V_OC_ losses that limit overall efficiency. The irregular, rough morphology of commercial CIGS surfaces prevents adequate coverage of perovskite materials, introducing interface recombination through incompletely passivated trap states at the perovskite/electron transport layer interface. Furthermore, deep-level defects at the perovskite surface and interface, including ion vacancies and dangling bonds formed during solution processing, trap charge carriers and cause significant recombination losses. A major bottleneck lies in energy losses stemming from strong interface recombination at the perovskite/ ETL interface, as C_60_, the commonly used ETL, contributes to nonradiative recombination due to interface defects and ionic migration [238, 260, 283, 284]. The FF and V_OC_ losses represent an additional barrier: the FF × V_OC_ value for perovskite–CIGS tandems reach only 60% of its theoretical limit, necessitating improvements in cell design for better charge carrier management and surface passivation. Efficiency challenges are also associated with voltage loss and phase segregation in the wide-bandgap perovskite top cell. Non-radiative recombination of charge carriers predominantly facilitated by defect states constitutes the primary loss mechanism in thin-film photovoltaic devices, largely stemming from disruptions in crystal periodicity at material interfaces [12, 26].

#### Manufacturing scalability and economic considerations

From a manufacturing perspective, monolithic 2 T tandems offer the highest efficiency potential, already exceeding 30% in the best demonstrations. However, they also impose the most stringent process-compatibility constraints. The perovskite top cell must be fabricated without damaging the underlying CIGS stack, and the integrated production line must accommodate materials with very different thermal, chemical, and solvent sensitivities. This raises process complexity and capital investment requirements. Mechanically stacked four-terminal tandems present a simpler alternative because the subcells can be processed independently. This relaxes current-matching constraints and eliminates the need for a recombination layer, thereby reducing interface process risk. The trade-off is lower efficiency due to parasitic absorption in transparent conductors and encapsulants, together with a potentially more complex module package. Nevertheless, the lower capital barrier and compatibility with existing CIGS and perovskite production lines make 4 T architectures attractive for near-term commercialization. Laminated tandem architecture occupies an intermediate position. Emerging designs based on transparent conductive adhesives, including metal-free TCA concepts, avoid some shunting risks while retaining a partially integrated form factor. Their conductivity must still be optimized carefully, but they offer a plausible middle ground between monolithic and mechanically stacked devices. Demonstrations at areas above 1 cm^2^ suggest that scalable fabrication routes are beginning to emerge.

Three-terminal (3 T) tandems occupy an intermediate position between monolithic 2 T and mechanically stacked 4 T architectures by introducing an additional electrical terminal while retaining a high degree of optical integration. This configuration can partially decouple the electrical operation of the two subcells, thereby relaxing the stringent current-matching requirement of 2 T tandems. For CIGS/perovskite tandems, a voltage-matched p–n–p configuration is particularly attractive because the polarity of the constituent subcells can enable 3 T operation. However, this architectural flexibility comes at the cost of increased device and process complexity. Additional contacts and electrical isolation must be incorporated without introducing excessive parasitic resistance or optical losses, while efficient lateral current transport and reliable module-level interconnection remain challenging. Moreover, the fabrication sequence must maintain compatibility between the CIGS and perovskite subcells while avoiding shunting and interfacial degradation. As a result, 3 T CIGS/perovskite tandems remain considerably less mature than their 2 T counterparts. Further advances in terminal design, lateral current collection, parasitic-loss minimization, and scalable interconnection will therefore be required to establish 3 T architectures as a practical alternative. Nevertheless, their ability to relax current-matching constraints while maintaining a more integrated structure makes 3 T tandems a promising complementary pathway between the efficiency potential of 2 T devices and the fabrication flexibility of 4 T configurations.

Beyond the device-level bottlenecks discussed above, achieving clear progress toward economically relevant CIGS/perovskite tandems will require confronting several hurdles that extend past cell efficiency and stability metrics. A critical, often overlooked constraint is the supply-chain criticality of In and Ga, both concentrated by-products of Zn and Al refining with volatile pricing and limited recovery infrastructure [285, 286]; techno-economic roadmaps for this technology should therefore incorporate material criticality assessments and end-of-life recycling pathways alongside conventional levelized cost of electricity (LCOE) modeling [285, 287]. Relatedly, the reliance of record-efficiency CIGS absorbers on CdS buffer layers presents a regulatory and environmental liability under tightening restrictions on hazardous substances (RoHS-type restrictions), necessitating further development of Cd-free alternatives such as Zn(O,S) that remain thermally and chemically compatible with subsequent low-temperature perovskite deposition [288]. From a manufacturing standpoint, the cell-to-module efficiency gap driven by laser-scribing dead zones, TCO sheet-resistance losses, and declining yield at industrially relevant module areas (> 100 cm^2^) remains insufficiently characterized for this tandem architecture and is likely to be the dominant factor separating laboratory promise from fab-line economics [234]. Given that CIGS/perovskite tandems are unlikely to undercut Si/perovskite tandems on utility-scale $/W, economic viability will more plausibly hinge on deployment niches where CIGS's lightweight, flexible, and high specific-power characteristics confer a value-per-weight or value-per-area advantage, such as building-integrated photovoltaics, aerospace, and agrivoltaic applications; dedicated LCOE frameworks tailored to these use cases are presently lacking [289, 290]. Finally, inconsistent cross-laboratory efficiency reporting practices (e.g., spectral mismatch correction, aperture masking, and hysteresis treatment) and the absence of standardized life-cycle assessments for Pb-based In-Ga-Se/perovskite stacks continue to hinder reliable benchmarking and long-term bankability [291]. Addressing these interconnected material, manufacturing, and economic factors alongside the device-level improvements outlined above will be essential for translating laboratory-scale CIGS/perovskite tandem breakthroughs into commercial and environmentally sustainable photovoltaic technology.

#### Outstanding research gaps and priority areas

Several research gaps remain decisive. First, more direct and standardized methods are needed to quantify interface defect density and surface recombination velocity across different CIGS/perovskite interface combinations. Second, the mechanistic relationship between mechanical stress, defect formation, and long-term degradation needs deeper clarification, particularly under simultaneous illumination, thermal cycling, and humidity exposure. Third, field-reliability datasets extending beyond three years are still absent, limiting realistic lifetime projections and techno-economic modeling. Further, the field requires demonstrations of large-area monolithic tandems above 1 cm^2^ that combine efficiency above 28% with operational stability beyond 1000 h. Detailed cost–benefit comparisons of 2 T, 4 T, and laminated architectures are also needed for specific deployment scenarios. Finally, if environmental regulations tighten, lead-free tandem pathways may become more important, which would intensify the need for stable high-performance Sn-based or other non-toxic top cell materials.

#### Future outlook

- Bottom CIGS cell
  Atomically engineered interfaces for tandem CIGS: Future studies should focus on atomic-scale control of front and rear interfaces to optimize band alignment and suppress recombination in wide-bandgap CIGS tandem absorbers.
  Predictive and spatially controlled alkali engineering: Future research should focus on controlling the spatial distribution of alkali elements (Na, K, Rb, and Cs) within CIGS absorbers to achieve uniform defect passivation, improved carrier homogeneity, and enhanced long-term device stability.
  Precise control of secondary phases and surface stoichiometry: Optimizing ordered vacancy compounds and alkali-induced surface phases will be critical for balancing defect passivation with efficient carrier transport.
  Data-driven defect engineering and multiscale modeling: Combining machine learning, density functional theory, and device simulations can accelerate predictive design of defect-controlled CIGS/perovskite tandem solar cells.
- Top perovskite cell
  Future advancements in wide-bandgap perovskites should prioritize suppressing deep-level defect formation, photo-induced halide segregation, and ionic migration through multi-cation/anion compositional engineering, pseudohalide incorporation, and GB-selective passivation to minimize SRH recombination and V_OC_ deficit in tandem devices.
  Advanced interface engineering strategies involving a combination of SAMs, dipole-inducing molecular interlayers, 2D/3D heterostructures, and ALD-grown ultrathin oxides are required to optimize interface band alignment, suppress interface trap states, reduce carrier accumulation, and stabilize coupled ionic-electronic transport at buried interfaces.
  The realization of stable high-efficiency CIGS/perovskite tandem solar cells will depend on low-temperature interface-compatible processing, operando characterization of defect evolution, and predictive computational frameworks integrating first-principles modeling, machine learning, and interface energetics optimization for scalable tandem-device integration.
- 2 T CIGS/perovskite tandem solar cells
  Interface and Interconnecting Layer Engineering: Future research must develop thermally robust passivation compounds that resist thermal desorption under simultaneous illumination and elevated temperature stresses, while designing lossless interconnecting layers that eliminate parasitic optical and electrical losses inherent in conventional metal-based recombination layers.
  Environmental Durability and Manufacturing Scale-Up: Addressing fundamental challenges in long-term stability through advanced encapsulation techniques, lead-free alternatives, and moisture/UV resistance improvements is critical for commercialization. Mid- and long-term strategies (3–10 years) should focus on roll-to-roll manufacturing with ≥ 90% capacity utilization and development of low-cost alternative materials to reduce production costs and environmental footprint.
  Furthermore, the key scientific bottlenecks, technological roadmap, and associated performance targets have been discussed in detail in the following section and Table 5.

**Table 5:** Technological roadmap and performance targets for 2 T CIGS/perovskite tandem solar cells to achieve efficiencies beyond 33% with long-term operational stability

Timeframe	Milestone	Target PCE
Near-term (1–2 yrs)	Optimized wide-gap perovskite + low-damage TCO	31–32%
Mid-term (3–5 yrs)	Improved current matching + interconnect optimization	~ 33%
Long-term (5+ yrs)	Stable wide-gap perovskite + industrially compatible CIGS + module-level stability certification	> 33%, with IEC-equivalent stability qualification

## Key scientific bottlenecks and associated performance targets

1. Bandgap and current-matching optimization
  Challenge: The wide-bandgap perovskite top cell (currently ~ 1.63–1.68 eV in most highly efficient devices) is not yet optimal for the graded CIGS bottom cell (~ 1.0–1.15 eV effective gap). Subcell current mismatch currently limits J_SC_ to ~ 19–20 mA cm^−2^.
  Target: Identify a top cell composition with sufficient halide (Br) content while suppressing light-induced phase segregation, to push matched J_SC_ to ~ 21–22 mA cm^−2^ without sacrificing V_OC_.
2. Wide-bandgap perovskite stability (halide segregation and defect density)
  Challenge: Higher-Br perovskites needed for optimal bandgap pairing are more prone to the Hoke effect and have historically shown 100–150 mV higher non-radiative V_OC_ deficits than narrower-gap compositions.
  Target: V_OC_ deficit < 400 mV for the wide-gap subcell (vs. current ~450–500 mV), via A-site cation engineering (Cs/FA/MA mixtures), surface passivation (2D capping layers, alkylammonium salts), and reduced bulk trap density (< 10^15^ cm^−3^).
3. Recombination junction / ICL
  Challenge: The ICL between the perovskite and CIGS subcells must simultaneously provide ohmic recombination, optical transparency, and process compatibility (avoiding perovskite degradation from subsequent high-temperature or sputtering steps).
  Target: < 0.5 mA cm^−2^ parasitic optical loss in the interconnect stack; contact resistance < 1 Ω·cm^2^.
4. Transparent top electrode deposition
  Challenge: Sputter damage to the underlying perovskite during transparent ITO/IZO electrode deposition remains a major challenge.
  Target: Damage-free TCO deposition (via buffer layers or low-damage RF sputtering/ALD) enabling FF > 80% at the tandem level (current champion devices: ~ 78–79%).
5. Long-term operational stability
  Challenge: Combined thermal (CIGS back-contact/Mo interactions), moisture, UV, and ion-migration stresses are not yet characterized under IEC 61215-equivalent protocols for this specific tandem architecture.
  Targets: T80 > 1000 h under 1-sun illumination at 85 °C (ISOS-L-2 or equivalent), pass damp heat (85 °C/85% RH, 1000 h) with <5% relative efficiency loss, UV-preconditioning stability without significant PCE loss (ISOS-L-1)

## Data Availability

This review did not generate or analyze any new datasets. All data and materials referenced herein are obtained from previously published studies and publicly available scientific literature.
